# Mutation Detection in an Antibody-Producing Chinese Hamster Ovary Cell Line by Targeted RNA Sequencing

**DOI:** 10.1155/2016/8356435

**Published:** 2016-03-20

**Authors:** Siyan Zhang, Jason D. Hughes, Nicholas Murgolo, Diane Levitan, Janice Chen, Zhong Liu, Shuangping Shi

**Affiliations:** ^1^Biologics & Vaccines, Merck Research Laboratories, Kenilworth, NJ 07033, USA; ^2^Biology & Genetics Informatics, Merck Research Labs IT, Merck & Co, Boston, MA 02115, USA; ^3^Discovery Pharmacogenomics, Merck Research Laboratories, Kenilworth, NJ 07033, USA

## Abstract

Chinese hamster ovary (CHO) cells have been used widely in the pharmaceutical industry for production of biological therapeutics including monoclonal antibodies (mAb). The integrity of the gene of interest and the accuracy of the relay of genetic information impact product quality and patient safety. Here we employed next-generation sequencing, particularly RNA-seq, and developed a method to systematically analyze the mutation rate of the mRNA of CHO cell lines producing a mAb. The effect of an extended culturing period to mimic the scale of cell expansion in a manufacturing process and varying selection pressure in the cell culture were also closely examined.

## 1. Introduction

The development of next-generation sequencing (NGS) technologies has greatly improved the efficiency of sequencing and contributed to the understanding of dynamic changes in gene expression [1]. With the maturation of NGS, its applications in biomedical research and drug discovery have greatly advanced the identification of disease related mutations and the development of molecules targeting the aberrantly expressed gene products [2–6]. Massively parallel cDNA sequencing (RNA-seq) has revolutionized transcriptomics studies compared to microarray technologies [7]. RNA-seq allows both qualitative and quantitative analysis of the expressed gene product at messenger RNA (mRNA) level with wide dynamic ranges and superior sensitivity [8].

Mammalian cell lines such as the Chinese hamster ovary (CHO) cells have been widely used in the production of recombinant therapeutic product including monoclonal antibodies [9, 10]. These cell lines are propagated extensively to reach large-scale production vessel. Production cell lines are generated by transfecting the host cells with a plasmid vector expressing the gene of interest (GOI) and a selection marker, followed by drug treatment and clone selection. During a large-scale manufacturing process, cells from a frozen bank need to be expanded multiple times to reach a final volume as large as 20,000 liters. The integrity of the GOI and the accurate flow of genetic information throughout this process are crucial to product quality. Traditionally, protein sequencing and mass spectrometry are used to characterize the final product for its consistency and homogeneity at the protein level [11]. DNA sequencing based on the Sanger or pyrosequencing method has also been used for sequence analysis of the mRNA (via cDNA) [12]. Although these mammalian host cells have a proven track record in consistently producing high-quality products, a potential threat is posed to the quality of the final product by the drug selection process, cloning procedures, and environmental stress over extended passaging conditions [13]. Product variants including point mutations could develop during the life cycle of the production cells. However, the extent of this risk has not been fully understood due to the limitations of traditional molecular biology tools mentioned above.

In this study, we explored the use of RNA-seq technology for the characterization of the mutation rate in a stably transfected CHO cell line expressing a recombinant monoclonal antibody (mAb) under extensive* in vitro* passaging. The goal is to identify and quantify mutations in a cell population at the transcript level under various culture conditions. We first carried out a feasibility study by mixing two slightly different mAb light chain cDNAs at different ratios and subjected the mixture samples to RNA-seq analysis. The detection limit of the mutation rate was determined by the feasibility study. Since mutation rate is presumably related to the length of passaging and the presence of potentially mitogenic selection reagents, such as methotrexate (MTX), we next cultured the CHO cell line continuously to reach an* in vitro* cell age of ~150 population doubling levels (PDLs). In parallel, increasing the dose of MTX was also evaluated for its impact on mutation rate. The method we developed in this study will be instrumental in defining the cell culture parameters to ensure consistent and reliable product quality.

## 2. Materials and Methods

### 2.1. Feasibility Study by cDNA Mixing

Two cell clones (A and B) expressing a human IgG with different light chain (LC) sequences were thawed from frozen banks and cultured in alpha-MEM (Gibco, Cat. 12561) containing 10% dialyzed fetal bovine serum (FBS, SAFC, Cat. 12015C) and 0.45% glucose (Sigma, Cat. G8769). Cells were passaged and expanded for RNA extraction. RNA extraction was performed using the RNeasy kit (Qiagen, Cat. 74104), and RNA was eluted in 50 *μ*L RNase-free water. RNA concentration was measured on NanoDrop Spectrophotometer (ND-1000, Thermo Scientific).

RT-PCR of IgG light chains was set up with 200 ng RNA per sample using the OneStep RT-PCR kit (Qiagen, Cat. 210212) in 50 *μ*L reaction volume. RT-PCR was run on the Applied Biosystems 2720 Thermal Cycler with incubation periods of 30 min at 50°C and 15 min at 95°C, 30 cycles of 30-second denaturing at 94°C, 30-second annealing at 62°C, and 2 min extension at 72°C, followed by final 10 min incubation at 72°C. cDNA was purified using the Qiaquick PCR Purification Kit (Qiagen, Cat. 28106) and eluted in 30 *μ*L EB buffer (10 mM Tris-Cl, pH 8.5). cDNA concentrations were measured on NanoDrop. The cDNA of clone B was mixed with cDNA of clone A at mixing ratios of 5%, 1%, 0.5%, 0.1%, 0.05%, and 0.01%. Triplicate samples of pure cDNA of clones A and B and each mixture were submitted to BGI for RNA-seq.

See Supplementary Information in Supplementary Material available online at http://dx.doi.org/10.1155/2016/8356435 for light chain and primer sequences.

### 2.2. cDNA Preparation from Cell Line under Different Culture Conditions (Main Study)

Clone A, derived from a single cell, was thawed from a frozen bank at about 14 PDLs since serum-free adaptation and cultured in Ex-cell ACF CHO medium C5467 (SAFC Cat. 86016C-1000 mL) with 4 mM L-glutamine (Gibco, Cat. 25030), 1x Trace Elements A (Cellgro Cat. 99-182-C1), and 1x Trace Elements B (Cellgro Cat. 99-175-C1). Cells after thawing were termed PDL 0 and around 1 million cells were pelleted and resuspended in 350 *μ*L RLT buffer with 1% beta-mercaptoethanol for RNA extraction. Cells were further passaged at 0.5 million/mL every 3-4 days in the presence of 0, 20, or 80 nM MTX (Sigma Cat. 8407), at 37°C and 7.5% CO_2_.

**Figure 1:**
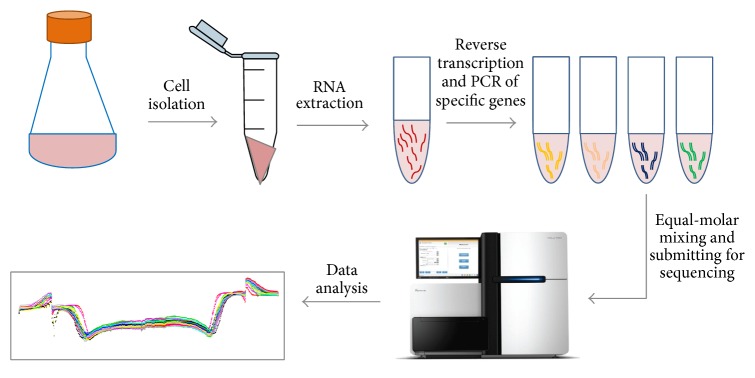
Experimental outline of RNA-seq studies of production CHO cell lines. The tested CHO cell lines expressing mAb were propagated in suspension. Cell pellets were isolated and RNA samples were subsequently extracted. Reverse transcription was performed on the RNA samples and certain genes of interest were amplified from cDNAs. After library preparation, the product was sequenced on Illumina HiSeq2000. Details of data analysis are described in Section 3.

At PDLs 0, 50, 100, and 150, 15 million cells were pelleted, divided into 3 aliquots upon lysis (except PDL 0 sample which was divided into replicates at RNA level) and RNA was extracted following Qiagen protocol (Qiagen RNeasy kit, Cat. 74104). Reverse transcription was performed with 200 ng RNA using the AccuScript High Fidelity RT-PCR kits (Agilent, Cat. 600180). The thermal program includes 5 min incubation at 65°C and cooling to room temperature for 5 min, followed by addition of 1 *μ*L of 100 mM dithiothreitol (DTT) and 1 *μ*L of AccuScript Reverse Transcriptase. The reaction was further incubated at 42°C for 30 min and stored at 4°C. Three separate reverse transcription reactions were performed for PDL 0 RNA to create replicates. cDNAs of heavy chain (HC), light chain (LC), dihydrofolate reductase (DHFR), and GAPDH were amplified via PCR using PfuUltra HF DNA polymerase (Agilent, Cat. 600380) and the following thermal cycle program: 1 min at 95°C, 30 cycles of 30 seconds at 95°C, 30 seconds at 64°C (62°C annealing was used for DHFR), and 3 min at 68°C, followed by a final 10 min incubation at 68°C. PCR products were purified using Qiaquick PCR Purification Kit (Qiagen, Cat. 28104). For each sample, equal-molar ratios of HC, LC, DHFR, and GAPDH were mixed to a total cDNA mass of 2.5 *μ*g and submitted for RNA-seq at BGI. The experimental procedure is outlined in Figure 1.

For the feasibility study, the amplified fragment for light chain corresponded precisely to the target sequence. In the main study, a slightly larger region was amplified for each target to ensure that the region of interest was outside the range of the PCR primers themselves. The references used for mapping were modified accordingly.

### 2.3. RNA-Seq

At BGI, cDNA was fragmented to an average fragment size of 170–180 bp using Covaris. On Thermomixer, these fragments were subjected to end-repair and the 3′ end was adenylated. Adaptors were ligated to the 3′ ends. The ligation products were purified on TAE-agarose gel, and ~14 rounds of PCR amplification were performed to enrich the purified cDNA template. For quality control, the library was validated on the Agilent Technologies 2100 Bioanalyzer and the ABI StepOnePlus Real-Time PCR System. Qualified libraries were sequenced on Illumina HiSeq2000 and 100 Mb clean sequence data were generated for each.

See Supplementary Information for details on sequences of primers and amplified regions. Analysis was performed excluding the regions corresponding to the PCR primers.

## 3. Results

### 3.1. Feasibility Study

cDNAs from two clones expressing light chain with closely related but slightly differing sequences were mixed in different ratios to assess the ability of NGS to quantitatively detect the fraction of mutant bases in a mixed population. The sequences chosen for this were each 714 bases long and differed in 46 positions. The sequence alignment is shown in Figure S1.

Detecting the fraction of sequence reads from a mixture of these clones is fundamentally different than detecting emerging mutations in cell culture in that one would not expect to find so many mutations emerging at once. In terms of the data analysis, the main impact is on the ability to map reads. For example, in the sequence between positions 80 and 120, there are more than a dozen sequence differences. By default, most short-read mappers will only map reads reliably when the error rate is less than around 5%. If sequences including mixtures of reads from clones A and B were mapped directly to clone A reference, some reads from clone B would not map at all to clone A reference. This would not be expected to happen in the real case of an emerging mutation at a single position. To address this issue, for the feasibility study, we map reads to a reference sequence that includes both clone A and clone B sequences, using BWA (https://github.com/lh3/bwa; version 0.7.0; Li H. and Durbin R. (2009) Fast and accurate short read alignment with Burrows-Wheeler transform. Bioinformatics, 25, 1754–1760. [PMID: 19451168]). BWA will output the single best alignment for each read in SAM format. For reads from regions where clones A and B differ, the alignment will indicate that the mapping was specific to reference A or B. For reads from regions where clones A and B do not differ, reads will be randomly assigned to one reference or the other. In order to obtain a mapping that is consistent with what we would expect to find in the real study if any one of the 46 mutations had occurred singly, we modify the mappings obtained in this way as follows. We replace all occurrences of the clone B sequence identifier in the SAM-formatted alignment files with the clone A identifier, and we ignore the trailing tag fields. Since there are no insertion or deletion differences between the two clones, the SAM file obtained in this way is perfectly consistent with what would have been obtained if the mutations had occurred separately. This procedure is equivalent to mapping reads to each of the clone sequences separately, determining which reference was a better fit and then translating the clone B alignments to become clone A alignments. In this case, that translation step is trivial since the two sequences differ only by substitutions. The key advantage of this approach over any single-reference mapping approach is that it eliminates the possibility of any edge effects or incorrectly induced insertions or deletions in the alignments in regions where the clones A and B sequences are significantly different. Had we used a more exhaustive approach such as a Smith-Waterman alignment of all reads to the clone A sequence, for example, the resulting alignments of reads from clone B that included significantly differing sections would have had small errors in alignment that would have confounded the analysis. Also, it is important to note that this modified alignment procedure is only relevant for the initial validation portion of this study.

Aside from this mapping difference, the analysis for the feasibility study is performed exactly as for the main study. Sequence data were received from BGI in FASTQ format. Adapters were removed using SeqPrep (https://github.com/jstjohn/SeqPrep; version 0.4; unpublished) and aligned to the reference sequence using BWA. Coverage across the light chain sequence for all samples is shown in Figure S2. The overall mapping rate across all experiments was very high, generally around 99%, and the reads aligned with a very low mismatch rate, typically around 0.2 mismatches per 90 bp read. This indicates that we had very little contamination in the experiment.

The SAMtools program “mpileup” (https://github.com/samtools/samtools; version 0.1.19; Li H.^*∗*^, Handsaker B.^*∗*^, Wysoker A., Fennell T., Ruan J., Homer N., Marth G., Abecasis G., and Durbin R. and 1000 Genome Project Data Processing Subgroup (2009) The Sequence alignment/map (SAM) format and SAMtools. Bioinformatics, 25, 2078-9. [PMID: 19505943]) was used along with custom scripts to extract for each position in the target region the counts of each base of A, C, G, and T, as well as the numbers of insertions and deletions. Insertions were counted according to the base immediately preceding the insertion regardless of what sequence was being inserted. Similarly, deletions were attributed to the base being deleted, regardless of how many bases were spanned by the overall deletion. These counts were stratified based on whether they were found from reads aligned in the forward or reverse directions. Bases with quality scores less than 15 were ignored in this analysis. This cutoff was selected to remove a minimum amount of data (typically 2–5% of bases), while eliminating the lowest quality bases, which are mainly those with reported base quality of two, indicating that the sequencer failed to call the base at the position. Within each experiment, for each position in each target sequence, a preferred orientation was determined based on which orientation gave rise to higher overall coverage. Only data from reads in the preferred orientation at each position was used to generate final results. Overall, this step has the impact of removing a small portion of very-low-quality data, at the cost of ignoring just under half of the overall sequence data, which has little impact on most positions.

This decision to use only data from reads in a preferred orientation is driven by the fact that some sequence contexts are problematic for sequencing (observed in a variety of targeted sequencing experiments; unpublished results). The problem may arise from any step in the process, from amplification to library prep to the sequencing itself. The issue is often found in regions that are G-rich. The reads on the G-rich strand will often have errors, while the reads from the other C-rich strand do not. In those cases, we find that the “better” strand usually has higher coverage, presumably because the sequencer was unable to generate acceptable reads from that direction and/or some of the base calls had quality scores below the threshold of 15. By applying a cutoff based on coverage, we are able to identify the “better” strand without explicitly biasing the analysis to lower-frequency results. For consistency, the strand choice is made once for each unit of analysis, the feasibility study, and the main study.

**Figure 2:**
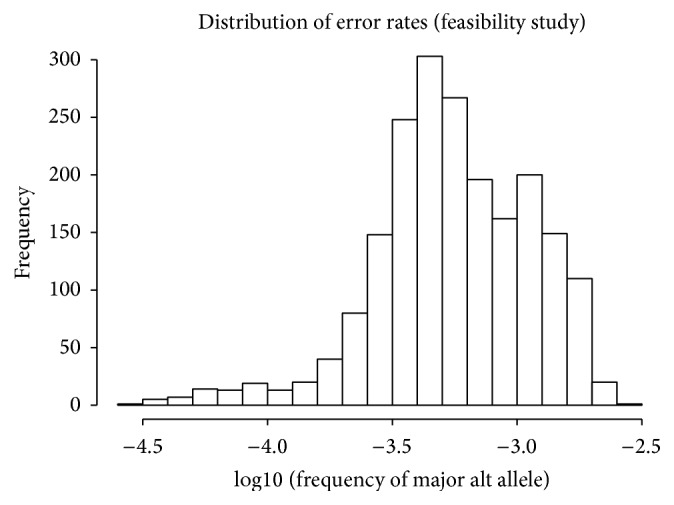
Distribution of error rates across all positions in light chain from the feasibility study. The most frequent alternate allele at each position is used to populate the figure.

Once the data have been processed to the counts of A, C, G, and T, indels and deletions for each position, we can determine the consensus sequence and the rate of occurrence for each possible alternate allele at each position. If we consider the data from the unmixed sample for clone A to be our reference, and any alternate allele observations to be errors, we find that the error rate across all possible positions, measured as the frequency of the most common alternate allele at each position, ranges from less than 0.01% to a high of 0.27%, with 99% of possible alternate alleles occurring at a rate of less than 0.2%. The full distribution is shown in Figure 2.

To assess the reproducibility of the data, we looked at the apparent error rates for each possible mutation using replicate experiments. Figure S3 shows plots of error versus error for two of the 100% clone A reference samples versus the third. The plot has a point for each possible base at each position, including the reference base. The reference base calls all hover near 1 when there are consensus base calls that all fit into the same pixel on the log-log plot. In this way, the plot focuses attention on the erroneous base calls. The red, green, and blue curves correspond to a difference in apparent mutation rate of 10%, 1%, and 0.1%, respectively. Using these plots, it is possible to quickly identify any outliers that might correspond to true mutations and to get an estimate of the overall noise level in the experiment.

For these samples, there are a few points very close to the blue 0.1% line, but none that actually cross it in either comparison. By contrast, when there is a true signal in the data set, data points are expected to be well outside this region. For example, if we take two of the 0.1% spiked controls and two of the 0.5% spiked controls and compare them to the 0% reference, we obtain the plots in Figure S4. The points corresponding to the true spiked-in mutations are colored red.

We will take the signal for each mutation in each spiked-in sample to be the difference between the average alternate allele rate observed in each of the three replicate spike-in samples and the average alternate allele rate observed for the corresponding mutation in the replicate reference samples. For each of these possible mutations, we will use a *t*-test to assess whether the difference between the two means is statistically significant. Given the small numbers of replicates involved, the *t*-test results will not be used aggressively, but rather as a filter to weed out spurious results (uncorrected *P* value cutoff of .01).

**Figure 3:**
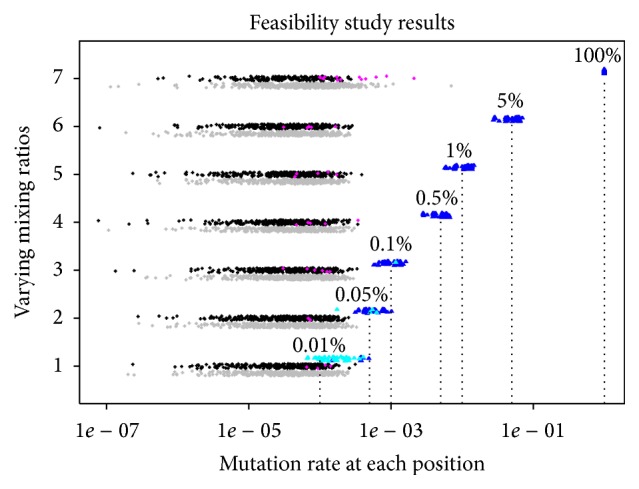
The seven horizontal bands of points correspond to experiments with mixing ratios of 0.01%, 0.05%, 0.1%, 0.5%, 1%, 5%, and 100%. There are points for each position in light chain for each sample sequenced. The *x*-axis corresponds to the apparent signal for each spiked-in sample. In order to include the negatives that result from this measurement on the log-scale plot, they are plotted as their absolute values, colored grey, and offset just below the other points. The points corresponding to the spiked-in mutations are colored blue and offset just above the other points. The light blue points did not meet the threshold for statistical significance. True-negative mutations that did meet the criteria for statistical significance are colored purple instead of black. All points have had a small amount of vertical jitter added. The jitter and offsets serve to allow visualization of the full distribution of points for the negative and positive controls.

The main results from the samples in the feasibility study are shown in Figure 3. We find that the estimates of mixing ratio are very accurate. The median signals at positive control sites for the 0.01%, 0.05%, 0.1%, 0.5%, 1%, and 5% spike-in experiments were 0.017%, 0.057%, 0.11%, 0.57%, 1.1%, and 5.3%, respectively. The range of signals was typically as much as ±2x, however. Certain sites have consistently lower or higher signal estimates across different spike-in levels, suggesting that the variability may be sequence-dependent and may not be corrected by additional sequencing.

All 46 true-positive mutations are observed with statistical significance for spike-in levels of 5%, 1%, and 0.5%. At the 0.1%, 0.05%, and 0.01% spike-in levels, 45/46, 42/46, and 10/46 of the mutations are observed. Across all control sites (true negative), 27 false positives were observed. The observed signal was less than 0.01% in most of those cases, and the highest signal observed was 0.03%. By contrast, for the positive control sites at the 0.1% spike-in level, the lowest observed excess signal was 0.0599%. Based on these observations, we set the following thresholds for mutation detection in the main study: excess mutation signal of more than 0.05% with a *P* value less than .01. In the feasibility study, these criteria would yield 45/46 true positives at the 0.1% spike-in level, with no false positives. The one false negative had an apparent signal of 0.12% but just barely missed the *P* value cutoff with a value of .012. Therefore, these settings are designed to be sufficient to detect (or rule out) mutations with a true signal of more than 0.1%.

It is worth noting here that had we been interested only in mutations at higher levels, the natural thresholds based on this feasibility study would always be around one-half of the desired mutation detection rate. That threshold would still allow perfect sensitivity for all 46 tested mutations, while minimizing the false positive rate.

### 3.2. Main Study

We found that the error profile for the main study was slightly different than that observed in the feasibility study. Overall, the error profile was better for the main study, with an average error rate over all possible substitutions and indels of .011%, versus .017% for the feasibility study.

**Figure 4:**
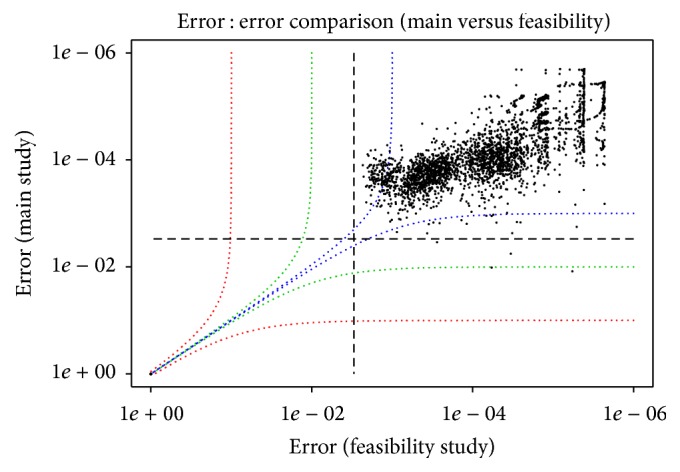
Comparison of a baseline sample from the main study versus a reference sample from the feasibility study, showing the rate of apparent error versus error for each possible alternate allele at each position. The dotted lines correspond to a mutation rate of 0.3%.

However, while there were no mutations with a background rate of more than 0.3% in the feasibility study, there were four such mutations in the main study, including two above the 1% level. The overall correspondence between the error rates was nevertheless quite good overall. See the error : error plot in Figure 4. More importantly, the error profiles for the main study samples compared to replicates within that study were very consistent. See the error : error plots for the reference samples in Figure S5.

**Figure 5:**
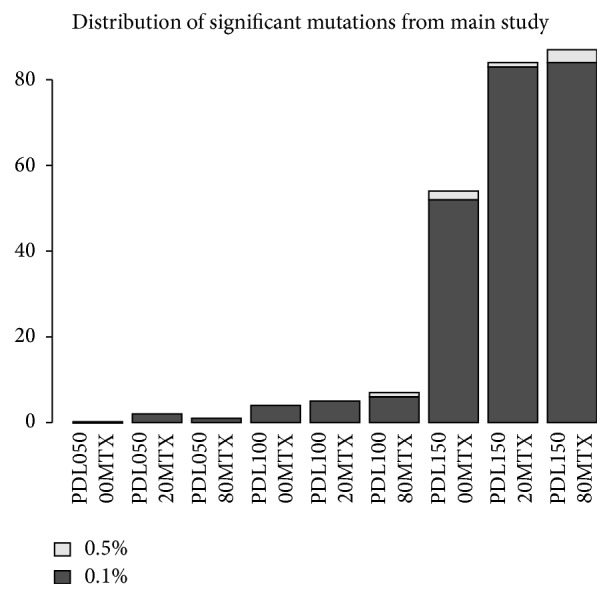
Histogram of counts of mutations meeting the threshold for detection of mutations at the 0.1% level for each experimental condition tested. Those mutations that also met the criteria for the 0.5% level are highlighted in light grey.

We proceeded with the analysis as described. Across all nine samples covering no MTX, 20 nM MTX, and 80 nM MTX at 50, 100, and 150 PDLs, 245 mutations met the criteria established in the feasibility study for the 0.1% level. These were unevenly distributed across the samples, biased strongly toward samples with larger PDLs. The distribution of mutations is shown in Figure 5. Also highlighted in this figure are those mutations that would have met the criteria for mutation detection at the 0.5% level. In total there were ten signals detected at that level.

**Figure 6:**
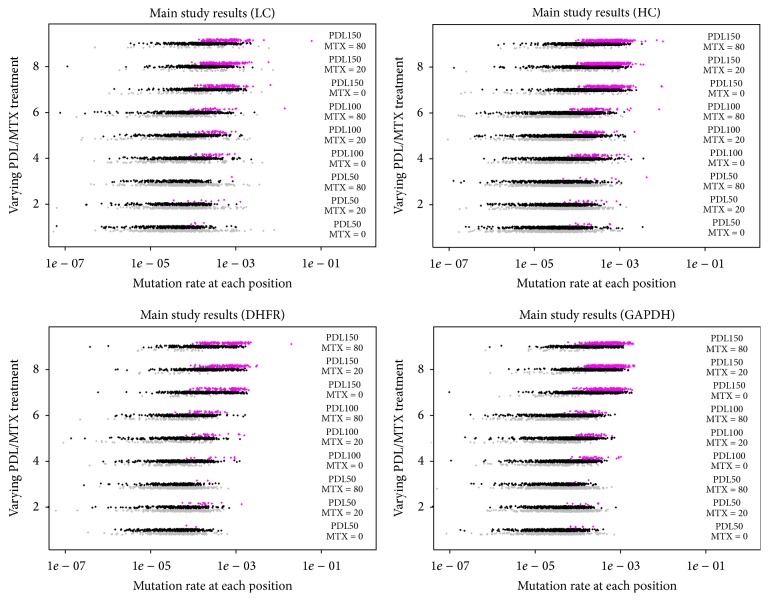
Four panels correspond to each of the four targets: light chain, heavy chain, GAPDH, and DHFR (clockwise from the top left). Each panel has points for each experimental condition, stratified vertically, exactly as done for the feasibility study (Figure 3). The coloring, jittering, and offsets for the points are also identical to Figure 3, except that there are no spike-in signals here, and hence no blue points. Positions meeting the criteria for significance (*t*-test *P* value <.01) are colored purple.

The same analysis was performed with identical settings for the other three targets in the experiment. The pattern of mutations was very similar in each case. The plots in Figure 6 show the apparent rate of mutation for all possible mutations in each of the four targets studied. In this more quantitative view, it is possible to see the full distribution of error rates across the study. While many mutations met the criteria for statistical significance (*t*-test *P* value <.01; points colored purple), the vast majority of those have a very low apparent mutation rate. Since we had only triplicate data, it was not possible to use a more stringent statistical cutoff. However, it is also possible to see some general trends in this view. Across all four targets, as the PDL increases, the distribution of apparent mutation rates shifts uniformly higher, for example. Presumably this reflects small, true shifts in the population accumulating over time, though few mutations met our criteria for detection. In terms of specific mutations meeting the criteria established for detection at the 0.5% level, the numbers of signals observed in light chain, heavy chain, DHFR, and GAPDH were 10, 17, 4, and 0, respectively. A table with all signals found across all four genes is included in the Supplementary Information.

## 4. Discussion

Here we explored using RNA-seq technology for the detection of emerging mutations in a CHO cell line producing a recombinant antibody during long-term culture. In the feasibility study, we established a high-confidence mutation level detection limit of 0.1%, which is significantly more sensitive than traditional molecular biology or protein characterization techniques. The detection limit of mutation by Sanger DNA sequencing is around 15–20% [14]. When comparing the feasibility study to the main study, we noticed that the background error profile revealed by sequencing replicates of the same biological sample can vary from batch to batch. Within each batch, the error profile at each position (whether arising from amplification, library prep, or sequencing itself) was very consistent. Therefore, a reference run should be included in each sequencing batch and used to assess variation within each batch. By considering each position to have an independent error profile, we can implicitly account for a variety of error sources without knowing exactly what contribution each source makes.

In the main study, we analyzed all three exogenous genes introduced by the expression vector, which were heavy chain and light chain of the mAb, and the DHFR selection marker. We also analyzed the house-keeping gene GAPDH as a representative host endogenous gene. As the study shows, the mutation rate displayed a clear increasing trend with extended culture passaging. And, in most cases, the mutation rate also increased in the presence of selection pressure (MTX). In the actual cell culture manufacturing process, the cell inoculum typically needs to be passaged for at least 30–40 PDLs starting from a frozen cell bank, and often in the presence of selection pressure such as MTX. Our experiments were designed to sufficiently cover this manufacturing window with respect to both process conditions. In Figure 6, there is a noticeable jump in the numbers of significant mutations (above 0.1%) starting at 150 PDLs. At the same time, up to 100 PDLs, only the sample treated with 80 nM MTX exhibited detectable mutations higher than 0.5%. No mutation above 0.5% was observed in the house-keeping gene GAPDH under any of the culture conditions. This indicates that increasing selection pressure and extending passaging period mainly affect the stability of the transgenes but have minimal effect on endogenous host genes, presumably due to the deleterious effect to the host. It is noteworthy that mutation rate can be described in two ways. The first is the number of mutations above the 0.1% detection limit across the entire gene fragment. And the second is the percentage of population that carries a specific point mutation. Both representations showed similar trend in our study.

On the molecular level, mutations identified in mRNA can be attributed to DNA template mutations [15], transcriptional errors [16, 17], or posttranscriptional modifications [8]. Understanding the mechanism behind individual mutations requires further characterization of all these possible factors, including DNA sequence analysis of the expression vector inserted into the genome. In addition, mutations detected by RNA-seq require confirmation by protein sequence analysis to assess their impact on product quality.

NGS technologies have played increasing roles in the development of cell culture production process and facilitated the understanding of the production cell line. There has not been a report on applying RNA sequencing to systematically analyze mutation rate during extended passaging of production CHO cells. Production cell line stability with respect to sequence integrity is crucial for the biopharmaceutical industry because cell lines carrying the intended transgene sequences are essential for product quality and patient safety. Here we have demonstrated that RNA-seq can help to ensure the accurate flow of genomic information to the final product. Although CHO cell lines developed with DHFR as the selection system are used as a model system in this study to characterize gene stability, the methods developed in this study should also be applicable for other production host cell lines and selection methodologies. The information generated should further stimulate investigation on the molecular mechanisms behind sequence variations in mRNA.

## Supplementary Material

Details on sequences of primers and amplified regions are provided in Supplementary Materials. In addition, mutations with signal above 0.1% in all four genes are summarized in a table. Figure S2 provides coverage of light chain sequence for all samples in the main study. Figure S3 to S5 provides error:error plots comparing the replicates of the reference sample from the feasibility study, the re-sults from two replicates of two different spiking levels (0.1% and 0.5%) from the feasibility study and the replicates of the baseline sample from the main study.
