# Supplementary material for: Mutation Detection in an Antibody-Producing Chinese Hamster Ovary Cell Line by Targeted RNA Sequencing
Source: Biomed Res Int. 2016 Mar 20;2016:8356435. doi: 10.1155/2016/8356435 (PMC4818804; doi:10.1155/2016/8356435)
Supplement: Supplementary file 1 — Details on sequences of primers and amplified regions are provided in Supplementary Materials. In addition, mutations with signal above 0.1% in all four genes are summarized in a table. Figure S2 provides coverage of light chain sequence for all samples in the main study. Figure S3 to S5 provides error:error plots comparing the replicates of the reference sample from the feasibility study, the re-sults from two replicates of two different spiking levels (0.1% and 0.5%) from the feasibility study and the replicates of the baseline sample from the main study. [file 8356435.f1.docx]

# FIGURES

cloneA 1 ATGGCCCCTGTGCAGCTGCTGGGCCTGCTGGTGCTGTTCCTGCCTGCCATGCGGTGCGAGATCGTGCTGACCCAGTCCCCTGCCACCCTGTCCCTGAGCC 100

|||||||||||||||||||||||||||||||||||||||||||||||||||||||||||||||||||||||||||||||||....|||||.|..|||.||

cloneB 1 ATGGCCCCTGTGCAGCTGCTGGGCCTGCTGGTGCTGTTCCTGCCTGCCATGCGGTGCGAGATCGTGCTGACCCAGTCCCCTCTGTCCCTGCCTGTGACCC 100

cloneA 101 CTGGCGAGCGGGCTACCCTGAGCTGCAGAGCCTCCAAGGGCGTGTCCACCTCCGGCTACTCCTACCTGCACTGGTATCAGCAGAAGCCAGGCCAGGCCCC 200

|||||||||..||..||.|...||||.|.|||||||||||||||||||||||||||||||||||||||||||||||||.|||||||||.||||||.||||

cloneB 101 CTGGCGAGCCTGCCTCCATCTCCTGCCGGGCCTCCAAGGGCGTGTCCACCTCCGGCTACTCCTACCTGCACTGGTATCTGCAGAAGCCTGGCCAGTCCCC 200

cloneA 201 TCGGCTGCTGATCTACCTGGCCTCCTACCTGGAGTCCGGCGTGCCTGCCCGGTTCTCCGGCTCCGGAAGCGGCACCGACTTCACCCTGACCATCTCCTCC 300

.|.||||||||||||||||||||||||||||||||||||||||||||.||||||||||||||||||.||||||||||||||||||||||..||||||...

cloneB 201 CCAGCTGCTGATCTACCTGGCCTCCTACCTGGAGTCCGGCGTGCCTGACCGGTTCTCCGGCTCCGGCAGCGGCACCGACTTCACCCTGAAGATCTCCCGG 300

cloneA 301 CTGGAGCCTGAGGACTTCGCCGTGTACTACTGCCAGCACTCCCGGGACCTGCCTCTGACCTTTGGCGGCGGAACAAAGGTGGAGATCAAGCGTACGGTGG 400

.|||||.|.||||||.|.|.||||||||||||||||||||||||||||||||||||||||||.|||...||.||.|||.|||||||||||||||||||||

cloneB 301 GTGGAGGCCGAGGACGTGGGCGTGTACTACTGCCAGCACTCCCGGGACCTGCCTCTGACCTTCGGCCAGGGCACCAAGCTGGAGATCAAGCGTACGGTGG 400

cloneA 401 CCGCTCCTTCCGTGTTCATCTTCCCTCCCTCCGACGAGCAGCTGAAGTCCGGCACCGCCTCCGTGGTGTGCCTGCTGAACAACTTCTACCCTCGGGAGGC 500

||||||||||||||||||||||||||||||||||||||||||||||||||||||||||||||||||||||||||||||||||||||||||||||||||||

cloneB 401 CCGCTCCTTCCGTGTTCATCTTCCCTCCCTCCGACGAGCAGCTGAAGTCCGGCACCGCCTCCGTGGTGTGCCTGCTGAACAACTTCTACCCTCGGGAGGC 500

cloneA 501 CAAGGTGCAGTGGAAGGTGGACAATGCCCTGCAGTCCGGCAACTCCCAGGAATCCGTCACCGAGCAGGACTCCAAGGACAGCACCTACTCCCTGTCCAGC 600

|||||||||||||||||||||||||||||||||||||||||||||||||||||||||||||||||||||||||||||||||||||||||||||||||..|

cloneB 501 CAAGGTGCAGTGGAAGGTGGACAATGCCCTGCAGTCCGGCAACTCCCAGGAATCCGTCACCGAGCAGGACTCCAAGGACAGCACCTACTCCCTGTCCTCC 600

cloneA 601 ACCCTGACACTGTCCAAGGCCGACTACGAGAAGCACAAGGTGTACGCCTGCGAGGTGACCCACCAGGGCCTGTCCAGCCCTGTGACCAAGTCCTTCAACC 700

||||||||.|||||||||||||||||||||||||||||||||||||||||||||||||||||||||||||||||||||||||||||||||||||||||||

cloneB 601 ACCCTGACCCTGTCCAAGGCCGACTACGAGAAGCACAAGGTGTACGCCTGCGAGGTGACCCACCAGGGCCTGTCCAGCCCTGTGACCAAGTCCTTCAACC 700

cloneA 701 GGGGCGAGTGCTGA 714

||||||||||||||

cloneB 701 GGGGCGAGTGCTGA 714

Figure S1. Pairwise sequence alignment of the two clones used in the feasibility study. They differ at 46 positions.


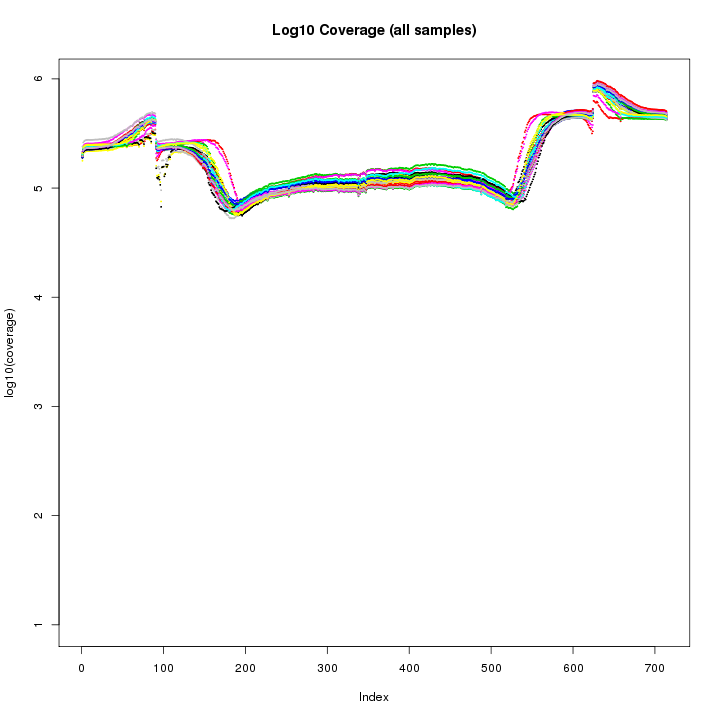


Figure S2. Coverage of light chain sequence for all samples in the main study.


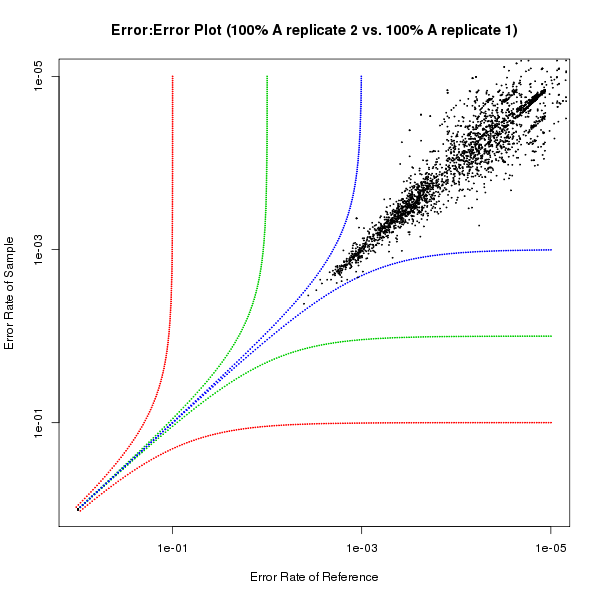

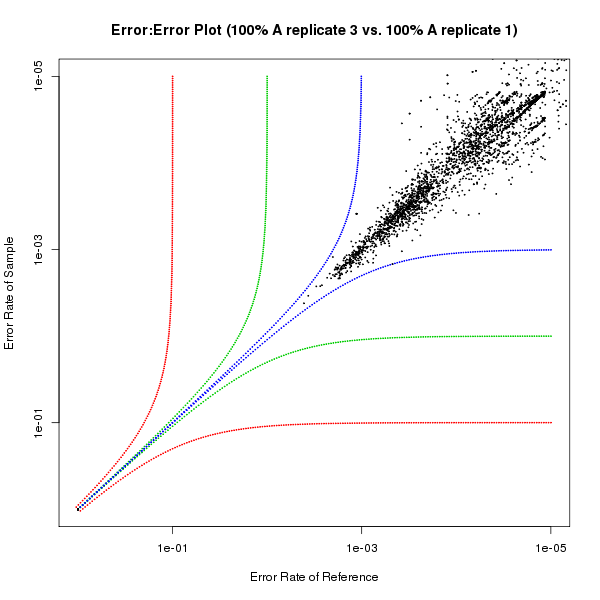


Figure S3. Error:error plots comparing the replicates of the reference sample from the feasibility study. Plots include a point for each possible mutation in light chain, with the axes corresponding to the apparent error rates for each replicates. The red, green, and blue lines correspond to differences in mutation rate of 10%, 1%, and 0.1%, respectively.


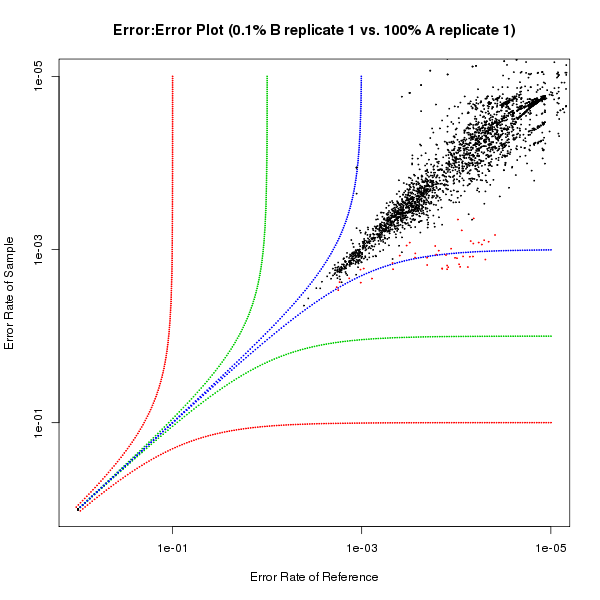

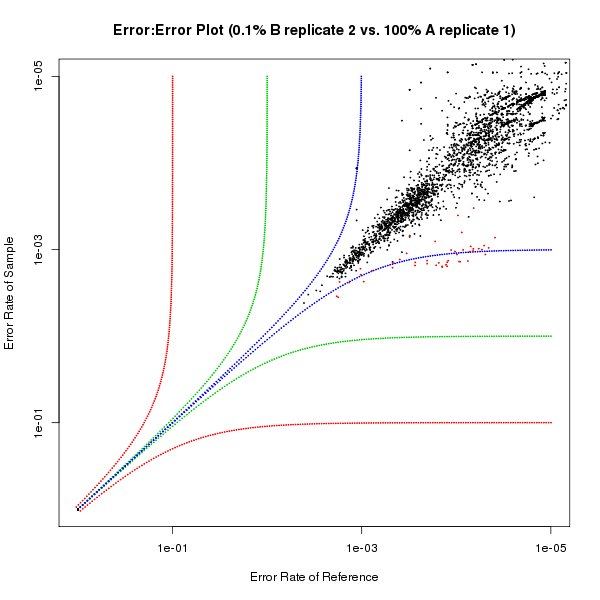

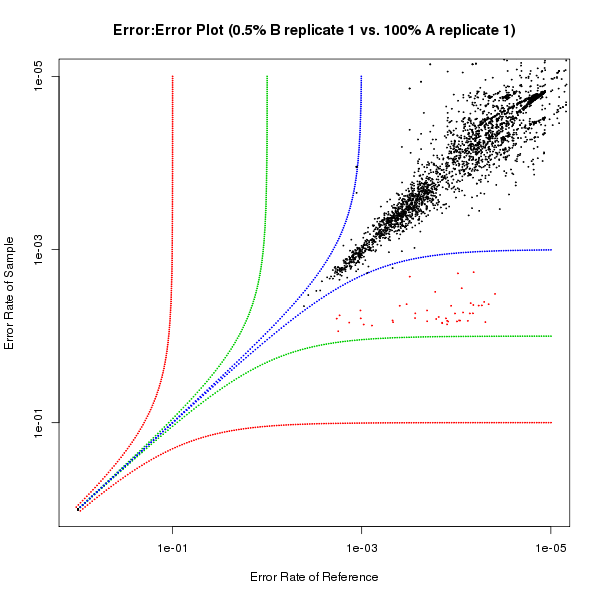

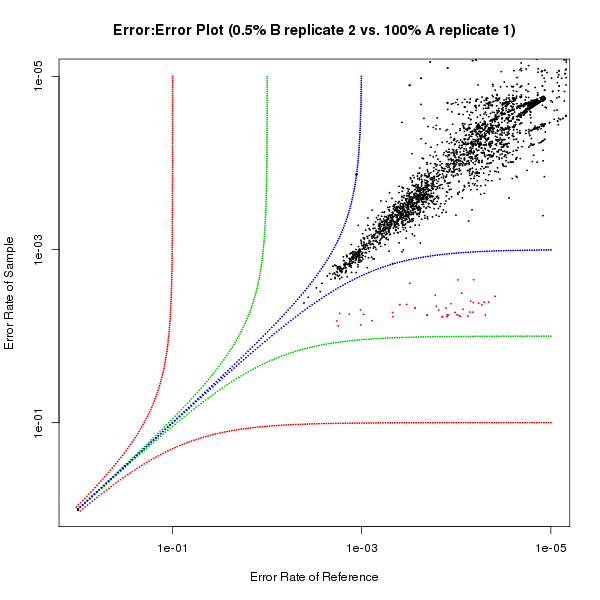


Figure S4. Error:error plots showing the results from two replicates of two different spiking levels (0.1% and 0.5%) from the feasibility study. See Fig. S3 for overall plot description. The points corresponding to the true mutations are colored purple.


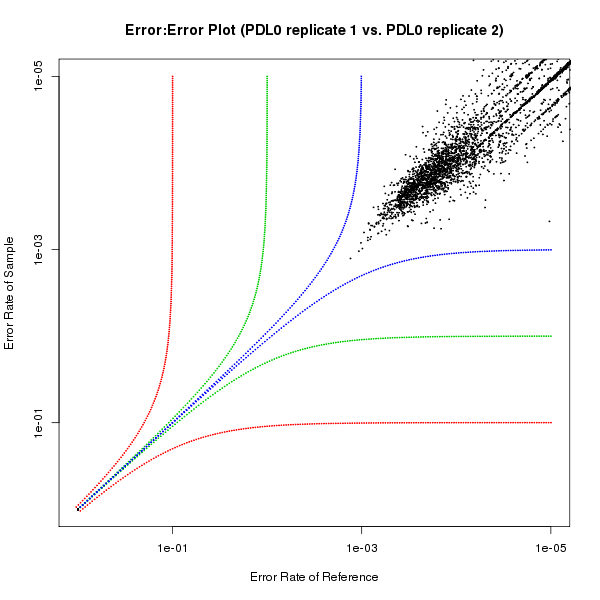

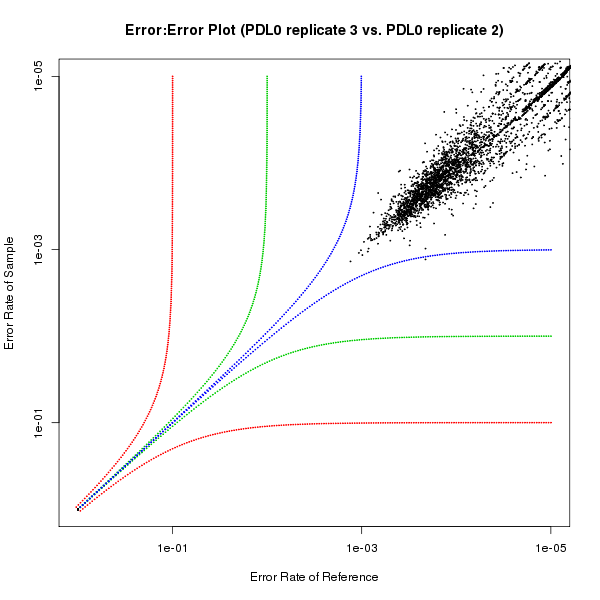


Figure S5. Error:error plot for replicates of the baseline sample from the main study. See Fig. S3 for details.

# PRIMERS AND AMPLIFIED GENE SEQUENCES

| **Primers** | **Sequence** |
| --- | --- |
| Light Chain _Fwd (feasibility) | ATGGCCCCTGTGCAGCTGCTG |
| Light Chain_Rev (feasibility) | TCAGCACTCGCCCCGGTTGAA |
| Light Chain_Fwd | CCACAGGCTTAAGAATTCCTCGA |
| Light Chain_Rev | GGGGATCTGGATCCCTCCC |
| Heavy Chain_Fwd | CCACAGGCTTAAGCTCGAGG |
| Heavy Chain_Rev | GGGGGATCTGGATCCATCG |
| DHFR_Fwd | CGCCAAACTTGACGGCA |
| DHFR_Rev | CCAGCAAAAGTCCCATGGTC |
| GAPDH_Fwd | TCCCTGTTCTAGAGACAGCCG |
| GAPDH_Rev | GGGTGCAGTGAACTTTATTGATGG |

**Light chain A (feasibility study)**

ATGGCCCCTGTGCAGCTGCTGGGCCTGCTGGTGCTGTTCCTGCCTGCCATGCGGTGCGAGATCGTGCTGACCCAGTCCCCTGCCACCCTGTCCCTGAGCCCTGGCGAGCGGGCTACCCTGAGCTGCAGAGCCTCCAAGGGCGTGTCCACCTCCGGCTACTCCTACCTGCACTGGTATCAGCAGAAGCCAGGCCAGGCCCCTCGGCTGCTGATCTACCTGGCCTCCTACCTGGAGTCCGGCGTGCCTGCCCGGTTCTCCGGCTCCGGAAGCGGCACCGACTTCACCCTGACCATCTCCTCCCTGGAGCCTGAGGACTTCGCCGTGTACTACTGCCAGCACTCCCGGGACCTGCCTCTGACCTTTGGCGGCGGAACAAAGGTGGAGATCAAGCGTACGGTGGCCGCTCCTTCCGTGTTCATCTTCCCTCCCTCCGACGAGCAGCTGAAGTCCGGCACCGCCTCCGTGGTGTGCCTGCTGAACAACTTCTACCCTCGGGAGGCCAAGGTGCAGTGGAAGGTGGACAATGCCCTGCAGTCCGGCAACTCCCAGGAATCCGTCACCGAGCAGGACTCCAAGGACAGCACCTACTCCCTGTCCAGCACCCTGACACTGTCCAAGGCCGACTACGAGAAGCACAAGGTGTACGCCTGCGAGGTGACCCACCAGGGCCTGTCCAGCCCTGTGACCAAGTCCTTCAACCGGGGCGAGTGCTGA

**Light chain B (feasibility study)**

ATGGCCCCTGTGCAGCTGCTGGGCCTGCTGGTGCTGTTCCTGCCTGCCATGCGGTGCGAGATCGTGCTGACCCAGTCCCCTCTGTCCCTGCCTGTGACCCCTGGCGAGCCTGCCTCCATCTCCTGCCGGGCCTCCAAGGGCGTGTCCACCTCCGGCTACTCCTACCTGCACTGGTATCTGCAGAAGCCTGGCCAGTCCCCCCAGCTGCTGATCTACCTGGCCTCCTACCTGGAGTCCGGCGTGCCTGACCGGTTCTCCGGCTCCGGCAGCGGCACCGACTTCACCCTGAAGATCTCCCGGGTGGAGGCCGAGGACGTGGGCGTGTACTACTGCCAGCACTCCCGGGACCTGCCTCTGACCTTCGGCCAGGGCACCAAGCTGGAGATCAAGCGTACGGTGGCCGCTCCTTCCGTGTTCATCTTCCCTCCCTCCGACGAGCAGCTGAAGTCCGGCACCGCCTCCGTGGTGTGCCTGCTGAACAACTTCTACCCTCGGGAGGCCAAGGTGCAGTGGAAGGTGGACAATGCCCTGCAGTCCGGCAACTCCCAGGAATCCGTCACCGAGCAGGACTCCAAGGACAGCACCTACTCCCTGTCCTCCACCCTGACCCTGTCCAAGGCCGACTACGAGAAGCACAAGGTGTACGCCTGCGAGGTGACCCACCAGGGCCTGTCCAGCCCTGTGACCAAGTCCTTCAACCGGGGCGAGTGCTGA

**Heavy Chain**

CCACAGGCTTAAGCTCGAGGCCGCCACCATGGCCGTGCTGGGCCTGCTGTTCTGCCTGGTGACCTTCCCTTCCTGCGTGCTGTCCCAGGTGCAGCTGGTGCAGTCCGGCGTGGAGGTGAAGAAGCCTGGCGCCTCCGTCAAGGTGTCCTGTAAGGCCTCCGGCTACACCTTCACCAACTACTACATGTACTGGGTGCGGCAGGCCCCAGGCCAGGGACTGGAGTGGATGGGCGGCATCAACCCTTCCAACGGCGGCACCAACTTCAACGAGAAGTTCAAGAACCGGGTGACCCTGACCACCGACTCCTCCACCACAACCGCCTACATGGAACTGAAGTCCCTGCAGTTCGACGACACCGCCGTGTACTACTGCGCCAGGCGGGACTACCGGTTCGACATGGGCTTCGACTACTGGGGCCAGGGCACCACCGTGACCGTGTCCTCCGCTAGCACCAAGGGCCCTTCCGTGTTCCCTCTGGCCCCTTGCTCCCGGTCCACCTCCGAGTCCACCGCCGCTCTGGGCTGTCTGGTGAAGGACTACTTCCCTGAGCCTGTGACCGTGAGCTGGAACTCTGGCGCCCTGACCTCCGGCGTGCACACCTTCCCTGCCGTGCTGCAGTCCTCCGGCCTGTACTCCCTGTCCTCCGTGGTGACCGTGCCTTCCTCCTCCCTGGGCACCAAGACCTACACCTGCAACGTGGACCACAAGCCTTCCAACACCAAGGTGGACAAGCGGGTGGAGTCCAAGTACGGCCCTCCTTGCCCTCCCTGCCCTGCCCCTGAGTTCCTGGGCGGACCCTCCGTGTTCCTGTTCCCTCCTAAGCCTAAGGACACCCTGATGATCTCCCGGACCCCTGAGGTGACCTGCGTGGTGGTGGACGTGTCCCAGGAAGATCCTGAGGTCCAGTTCAATTGGTACGTGGATGGCGTGGAGGTGCACAACGCCAAGACCAAGCCTCGGGAGGAACAGTTCAACTCCACCTACCGGGTGGTGTCTGTGCTGACCGTGCTGCACCAGGACTGGCTGAACGGCAAGGAATACAAGTGCAAGGTCAGCAACAAGGGCCTGCCCTCCTCCATCGAGAAAACCATCTCCAAGGCCAAGGGCCAGCCTCGCGAGCCTCAGGTGTACACCCTGCCTCCTAGCCAGGAAGAGATGACCAAGAATCAGGTGTCCCTGACATGCCTGGTGAAGGGCTTCTACCCTTCCGATATCGCCGTGGAGTGGGAGAGCAACGGCCAGCCAGAGAACAACTACAAGACCACCCCTCCTGTGCTGGACTCCGACGGCTCCTTCTTCCTGTACTCCAGGCTGACCGTGGACAAGTCCCGGTGGCAGGAAGGCAACGTCTTTTCCTGCTCCGTGATGCACGAGGCCCTGCACAACCACTACACCCAGAAGTCCCTGTCCCTGTCTCTGGGCAAGTGAATCGATGGATCCAGATCCCCC

**Light Chain** (main study, same coding sequence as Light Chain A in feasibility study, but primers were designed to amplify a region bigger than the coding region)

CCACAGGCTTAAGAATTCCTCGAGGCCGCCACCATGGCCCCTGTGCAGCTGCTGGGCCTGCTGGTGCTGTTCCTGCCTGCCATGCGGTGCGAGATCGTGCTGACCCAGTCCCCTGCCACCCTGTCCCTGAGCCCTGGCGAGCGGGCTACCCTGAGCTGCAGAGCCTCCAAGGGCGTGTCCACCTCCGGCTACTCCTACCTGCACTGGTATCAGCAGAAGCCAGGCCAGGCCCCTCGGCTGCTGATCTACCTGGCCTCCTACCTGGAGTCCGGCGTGCCTGCCCGGTTCTCCGGCTCCGGAAGCGGCACCGACTTCACCCTGACCATCTCCTCCCTGGAGCCTGAGGACTTCGCCGTGTACTACTGCCAGCACTCCCGGGACCTGCCTCTGACCTTTGGCGGCGGAACAAAGGTGGAGATCAAGCGTACGGTGGCCGCTCCTTCCGTGTTCATCTTCCCTCCCTCCGACGAGCAGCTGAAGTCCGGCACCGCCTCCGTGGTGTGCCTGCTGAACAACTTCTACCCTCGGGAGGCCAAGGTGCAGTGGAAGGTGGACAATGCCCTGCAGTCCGGCAACTCCCAGGAATCCGTCACCGAGCAGGACTCCAAGGACAGCACCTACTCCCTGTCCAGCACCCTGACACTGTCCAAGGCCGACTACGAGAAGCACAAGGTGTACGCCTGCGAGGTGACCCACCAGGGCCTGTCCAGCCCTGTGACCAAGTCCTTCAACCGGGGCGAGTGCTGAAGGGAGGGATCCAGATCCCC

**DHFR**

CGCCAAACTTGACGGCAATCCTAGCGTGAAGGCTGGTAGGATTTTATCCCCGCTGCCATCATGGTTCGACCATTGAACTGCATCGTCGCCGTGTCCCAAAATATGGGGATTGGCAAGAACGGAGACCTACCCTGGCCTCCGCTCAGGAACGAGTTCAAGTACTTCCAAAGAATGACCACAACCTCTTCAGTGGAAGGTAAACAGAATCTGGTGATTATGGGTAGGAAAACCTGGTTCTCCATTCCTGAGAAGAATCGACCTTTAAAGGACAGAATTAATATAGTTCTCAGTAGAGAACTCAAAGAACCACCACGAGGAGCTCATTTTCTTGCCAAAAGTTTGGATGATGCCTTAAGACTTATTGAACAACCGGAATTGGCAAGTAAAGTAGACATGGTTTGGATAGTCGGAGGCAGTTCTGTTTACCAGGAAGCCATGAATCAACCAGGCCACCTCAGACTCTTTGTGACAAGGATCATGCAGGAATTTGAAAGTGACACGTTTTTCCCAGAAATTGATTTGGGGAAATATAAACTTCTCCCAGAATACCCAGGCGTCCTCTCTGAGGTCCAGGAGGAAAAAGGCATCAAGTATAAGTTTGAAGTCTACGAGAAGAAAGACTAACAGGAAGATGCTTTCAAGTTCTCTGCTCCCCTCCTAAAGCTATGCATTTTTATAAGACCATGGGACTTTTGCTGG

**GAPDH**

TCCCTGTTCTAGAGACAGCCGCATCTTTCCGTGCAGTGCCAGCCTCGCTCCGGAGACGCAATGGTGAAGGTCGGCGTGAACGGATTTGGCCGTATTGGACGCCTGGTTACCAGGGCTGCCTTCACTTCTGGCAAAGTGGAAGTTGTTGCCATCAATGACCCCTTCATTGACCTCAACTACATGGTCTACATGTTCCAGTATGACTCTACCCATGGCAAGTTCAAAGGCACAGTCAAGGCTGAGAATGGAAAGCTTGTCATCAACGGGAAGGCCATCACCATCTTCCAGGAGCGAGATCCCGCCAACATCAAATGGGGTGATGCTGGCGCCGAGTATGTTGTGGAATCTACTGGCGTCTTCACCACCATGGAGAAGGCTGGGGCCCACTTGAAGGGCGGGGCCAAGAGGGTCATCATCTCCGCCCCTTCTGCTGATGCCCCCATGTTTGTGATGGGTGTGAACCAAGACAAGTATGACAACTCCCTCAAGATTGTCAGCAATGCGTCCTGCACCACCAACTGCTTAGCCCCCCTGGCCAAGGTCATCCATGACAACTTTGGCATTGTGGAAGGACTCATGACCACGGTCCATGCCATCACTGCCACCCAGAAGACTGTGGATGGCCCCTCCGGGAAGCTGTGGCGTGATGGCCGTGGGGCTGCCCAGAACATCATCCCTGCATCCACTGGCGCTGCCAAGGCTGTGGGCAAAGTCATCCCAGAGCTGAACGGGAAGCTGACTGGCATGGCCTTCCGTGTTCCTACCCCCAACGTGTCCGTTGTGGATCTGACATGTCGCCTGGAGAAACCTGCCAAGTATGAGGACATCAAGAAGGTGGTGAAGCAGGCATCTGAGGGCCCACTGAAGGGCATCCTGGGCTACACCGAGGACCAGGTTGTCTCCTGCGACTTCAACAGTGACTCCCACTCTTCCACCTTTGATGCTGGGGCTGGCATTGCTCTCAATGACAACTTTGTAAAGCTCATTTCCTGGTATGACAATGAATTTGGCTACAGCAACAGAGTGGTGGACCTCATGGCCTACATGGCCTCCAAGGAGTAAGAAGCCCACCCTGGACCATCCACCCCAGCAAGGACTCGAGCAAGAGGGAGGCCCTGGCTGCTGAGCAGTCCCTGTCCAATAACCCCCACACCGATCATCTCCCTCACAGTTTCCATCCCAGACCCCCAGAATAAGGAGGGGCTTAGGGAGCCCTACTCTCTTGAATACCATCAATAAAGTTCACTGCACCC

# ALL MUTATIONS WITH SIGNAL ABOVE 0.1% (‘Pvalue’ is from a T-test comparing the apparent mutations signals from triplicate experiments. ‘Signal’ is the average apparent mutation rate, calculated as the average difference between the mutation rate in the test samples and the reference samples. The alternate allele is listed as I or D for insertion and deletion, respectively.))

| Gene | Pos | Ref | Alt | Pvalue | Signal | PDL | MTX |
| --- | --- | --- | --- | --- | --- | --- | --- |
| LC | 36 | G | T | 0.00127072 | 0.00053672 | 150 | 80 |
| LC | 119 | C | I | 0.008552597 | 0.00063204 | 150 | 80 |
| LC | 126 | C | D | 0.009847146 | 0.00456065 | 150 | 80 |
| LC | 128 | T | I | 0.003192153 | 0.000570563 | 150 | 80 |
| LC | 146 | C | A | 0.009571852 | 0.00053188 | 150 | 80 |
| LC | 148 | A | C | 6.40E-05 | 0.001761187 | 150 | 80 |
| LC | 149 | C | A | 0.003283459 | 0.00077439 | 150 | 80 |
| LC | 151 | C | A | 0.005963719 | 0.00076051 | 150 | 80 |
| LC | 154 | A | C | 0.004774764 | 0.00050368 | 150 | 80 |
| LC | 165 | C | A | 0.004485504 | 0.000866713 | 150 | 80 |
| LC | 168 | C | A | 0.002431973 | 0.001122303 | 150 | 80 |
| LC | 174 | C | A | 9.26E-06 | 0.001317863 | 150 | 80 |
| LC | 175 | G | A | 0.000940742 | 0.00078143 | 150 | 80 |
| LC | 179 | C | A | 0.000928942 | 0.002033883 | 150 | 80 |
| LC | 181 | A | C | 0.006863704 | 0.00184892 | 150 | 80 |
| LC | 182 | C | A | 0.001123616 | 0.001689447 | 150 | 80 |
| LC | 183 | C | A | 0.000409006 | 0.00072156 | 150 | 80 |
| LC | 184 | T | A | 0.001584431 | 0.001869607 | 150 | 80 |
| LC | 186 | C | A | 0.002214045 | 0.00088448 | 150 | 80 |
| LC | 188 | G | A | 0.001020989 | 0.001280017 | 150 | 80 |
| LC | 194 | C | A | 9.42E-05 | 0.00211878 | 150 | 80 |
| LC | 197 | A | T | 0.001369451 | 0.00254691 | 150 | 80 |
| LC | 206 | G | T | 0.006414793 | 0.000570163 | 150 | 80 |
| LC | 224 | G | T | 0.000313103 | 0.001153523 | 150 | 80 |
| LC | 228 | G | T | 0.002756802 | 0.000788963 | 150 | 80 |
| LC | 265 | G | T | 0.008680506 | 0.000664017 | 150 | 80 |
| LC | 286 | T | G | 0.004016135 | 0.00129884 | 150 | 80 |
| LC | 292 | G | T | 0.000528285 | 0.000633503 | 150 | 80 |
| LC | 299 | G | T | 0.002458674 | 0.000776043 | 150 | 80 |
| LC | 321 | G | T | 0.001039249 | 0.00079565 | 150 | 80 |
| LC | 336 | G | T | 0.000908085 | 0.000961983 | 150 | 80 |
| LC | 343 | G | T | 0.005036706 | 0.000817157 | 150 | 80 |
| LC | 345 | G | T | 0.00790488 | 0.000971343 | 150 | 80 |
| LC | 346 | G | T | 0.000453316 | 0.000863327 | 150 | 80 |
| LC | 379 | G | T | 0.003688608 | 0.00066564 | 150 | 80 |
| LC | 385 | C | T | 0.004759262 | 0.00055912 | 150 | 80 |
| LC | 387 | T | C | 0.000152168 | 0.0005131 | 150 | 80 |
| LC | 390 | G | T | 0.003454585 | 0.00066103 | 150 | 80 |
| LC | 397 | G | T | 0.003279753 | 0.000784343 | 150 | 80 |
| LC | 398 | G | T | 0.001430399 | 0.00094817 | 150 | 80 |
| LC | 413 | T | G | 0.002859139 | 0.001692083 | 150 | 80 |
| LC | 415 | G | T | 0.000166307 | 0.000686257 | 150 | 80 |
| LC | 423 | G | T | 5.61E-05 | 0.000580687 | 150 | 80 |
| LC | 424 | C | T | 0.00529709 | 0.000529367 | 150 | 80 |
| LC | 426 | T | G | 0.001332099 | 0.000732443 | 150 | 80 |
| LC | 431 | T | G | 0.004384021 | 0.00078462 | 150 | 80 |
| LC | 453 | C | A | 0.004364681 | 0.00057431 | 150 | 80 |
| LC | 457 | C | A | 0.000265881 | 0.000689043 | 150 | 80 |
| LC | 461 | C | A | 0.004954054 | 0.00058749 | 150 | 80 |
| LC | 462 | C | A | 0.004938136 | 0.000513917 | 150 | 80 |
| LC | 463 | T | A | 0.000134114 | 0.05992227 | 150 | 80 |
| LC | 492 | C | A | 0.008427567 | 0.000619887 | 150 | 80 |
| LC | 494 | C | A | 0.005418246 | 0.000819673 | 150 | 80 |
| LC | 495 | C | A | 0.003659034 | 0.000544517 | 150 | 80 |
| LC | 499 | G | A | 0.007725639 | 0.000541597 | 150 | 80 |
| LC | 505 | C | A | 0.005542071 | 0.00053623 | 150 | 80 |
| LC | 508 | C | A | 0.000392643 | 0.000762937 | 150 | 80 |
| LC | 513 | C | A | 1.39E-06 | 0.00094816 | 150 | 80 |
| LC | 515 | A | C | 0.007099796 | 0.000853507 | 150 | 80 |
| LC | 519 | C | A | 0.005781023 | 0.000525247 | 150 | 80 |
| LC | 521 | A | C | 0.000326479 | 0.00204831 | 150 | 80 |
| LC | 523 | C | A | 0.008563747 | 0.00095903 | 150 | 80 |
| LC | 524 | C | A | 0.00147596 | 0.000739897 | 150 | 80 |
| LC | 525 | T | A | 8.68E-05 | 0.00058418 | 150 | 80 |
| LC | 533 | C | A | 0.006856305 | 0.001139 | 150 | 80 |
| LC | 552 | G | A | 0.000941294 | 0.00056219 | 150 | 80 |
| LC | 555 | C | T | 0.003185703 | 0.000578367 | 150 | 80 |
| LC | 561 | C | A | 0.002130079 | 0.000847583 | 150 | 80 |
| LC | 564 | G | A | 0.006705531 | 0.00055586 | 150 | 80 |
| LC | 575 | A | C | 0.002563818 | 0.00056497 | 150 | 80 |
| LC | 576 | C | T | 0.004584637 | 0.00104959 | 150 | 80 |
| LC | 579 | C | T | 0.005878425 | 0.00182076 | 150 | 80 |
| LC | 583 | G | T | 0.007205098 | 0.002142347 | 150 | 80 |
| LC | 586 | T | G | 4.98E-06 | 0.000981593 | 150 | 80 |
| LC | 590 | T | G | 0.001106175 | 0.00105888 | 150 | 80 |
| LC | 591 | C | T | 0.001958594 | 0.00056932 | 150 | 80 |
| LC | 593 | C | T | 0.005835219 | 0.000603807 | 150 | 80 |
| LC | 595 | G | T | 0.00689889 | 0.001025483 | 150 | 80 |
| LC | 596 | A | T | 0.00113191 | 0.00064551 | 150 | 80 |
| LC | 597 | G | T | 0.007221225 | 0.000909123 | 150 | 80 |
| LC | 600 | G | T | 0.008523136 | 0.00095933 | 150 | 80 |
| LC | 609 | G | T | 0.007828419 | 0.000562497 | 150 | 80 |
| LC | 720 | C | A | 0.004702885 | 0.000845037 | 150 | 80 |
| LC | 729 | C | A | 0.000988654 | 0.000790703 | 150 | 80 |
| LC | 731 | A | C | 0.000473623 | 0.000858603 | 150 | 80 |
| LC | 733 | C | A | 0.001263075 | 0.000599093 | 150 | 80 |
| LC | 747 | A | C | 0.000304547 | 0.0010279 | 150 | 80 |
| LC | 46 | C | T | 0.001986227 | 0.00069451 | 150 | 20 |
| LC | 126 | C | D | 0.008303396 | 0.005846333 | 150 | 20 |
| LC | 148 | A | C | 0.001313665 | 0.001581393 | 150 | 20 |
| LC | 149 | C | A | 0.004794686 | 0.000562227 | 150 | 20 |
| LC | 150 | C | A | 2.21E-06 | 0.000748623 | 150 | 20 |
| LC | 151 | C | A | 0.000465391 | 0.000802967 | 150 | 20 |
| LC | 159 | C | A | 0.009093097 | 0.00070926 | 150 | 20 |
| LC | 164 | C | A | 0.000224103 | 0.000891033 | 150 | 20 |
| LC | 165 | C | A | 0.003291131 | 0.00100436 | 150 | 20 |
| LC | 168 | C | A | 0.001989185 | 0.000873997 | 150 | 20 |
| LC | 174 | C | A | 0.000452451 | 0.001484547 | 150 | 20 |
| LC | 175 | G | A | 0.001118751 | 0.0006197 | 150 | 20 |
| LC | 179 | C | A | 3.83E-05 | 0.0016345 | 150 | 20 |
| LC | 181 | A | C | 9.92E-05 | 0.002349077 | 150 | 20 |
| LC | 182 | C | A | 0.00080624 | 0.001588233 | 150 | 20 |
| LC | 183 | C | A | 0.000673537 | 0.0007268 | 150 | 20 |
| LC | 184 | T | A | 0.005474024 | 0.00167832 | 150 | 20 |
| LC | 185 | C | A | 0.003224786 | 0.000882147 | 150 | 20 |
| LC | 186 | C | A | 0.003560914 | 0.000959623 | 150 | 20 |
| LC | 188 | G | A | 0.001518171 | 0.001429133 | 150 | 20 |
| LC | 189 | C | A | 0.005642173 | 0.001416143 | 150 | 20 |
| LC | 192 | C | A | 0.009218386 | 0.001150337 | 150 | 20 |
| LC | 193 | T | A | 0.001924963 | 0.00092767 | 150 | 20 |
| LC | 194 | C | A | 3.33E-05 | 0.001879947 | 150 | 20 |
| LC | 208 | T | G | 0.005134421 | 0.002074287 | 150 | 20 |
| LC | 216 | G | T | 0.00715091 | 0.000771403 | 150 | 20 |
| LC | 220 | C | T | 0.004132916 | 0.000702377 | 150 | 20 |
| LC | 223 | G | T | 0.006611048 | 0.000684993 | 150 | 20 |
| LC | 224 | G | T | 0.006567856 | 0.001479177 | 150 | 20 |
| LC | 228 | G | T | 0.0023132 | 0.00090372 | 150 | 20 |
| LC | 229 | G | T | 6.23E-05 | 0.000661623 | 150 | 20 |
| LC | 236 | G | T | 0.000413091 | 0.000757827 | 150 | 20 |
| LC | 252 | G | T | 5.02E-05 | 0.001071637 | 150 | 20 |
| LC | 261 | C | T | 0.008111017 | 0.000516753 | 150 | 20 |
| LC | 265 | G | T | 0.000246803 | 0.00066412 | 150 | 20 |
| LC | 267 | G | T | 0.000553203 | 0.000721953 | 150 | 20 |
| LC | 286 | T | G | 0.005783723 | 0.000979923 | 150 | 20 |
| LC | 292 | G | T | 5.05E-05 | 0.000824077 | 150 | 20 |
| LC | 299 | G | T | 4.62E-05 | 0.000529313 | 150 | 20 |
| LC | 336 | G | T | 0.00829597 | 0.00101154 | 150 | 20 |
| LC | 337 | G | T | 0.001494045 | 0.001222943 | 150 | 20 |
| LC | 340 | C | T | 9.83E-05 | 0.000520597 | 150 | 20 |
| LC | 343 | G | T | 0.006193145 | 0.000962947 | 150 | 20 |
| LC | 379 | G | T | 0.009288038 | 0.000653027 | 150 | 20 |
| LC | 385 | C | T | 0.000768063 | 0.000543973 | 150 | 20 |
| LC | 390 | G | T | 0.00031629 | 0.00050068 | 150 | 20 |
| LC | 397 | G | T | 0.006104164 | 0.00077806 | 150 | 20 |
| LC | 398 | G | T | 0.007105594 | 0.000982837 | 150 | 20 |
| LC | 413 | T | G | 0.002394546 | 0.00164728 | 150 | 20 |
| LC | 414 | G | T | 0.005744282 | 0.00052805 | 150 | 20 |
| LC | 415 | G | T | 0.002589075 | 0.00058222 | 150 | 20 |
| LC | 423 | G | T | 0.001746503 | 0.00063348 | 150 | 20 |
| LC | 426 | T | G | 0.002677418 | 0.000794337 | 150 | 20 |
| LC | 429 | G | T | 0.007443465 | 0.000503803 | 150 | 20 |
| LC | 457 | C | A | 0.009999483 | 0.000504423 | 150 | 20 |
| LC | 476 | T | C | 0.009212124 | 0.000509 | 150 | 20 |
| LC | 498 | G | A | 0.004761652 | 0.00052456 | 150 | 20 |
| LC | 507 | G | A | 0.009141068 | 0.00050558 | 150 | 20 |
| LC | 513 | C | A | 0.008000237 | 0.00069801 | 150 | 20 |
| LC | 515 | A | C | 0.004124287 | 0.00094184 | 150 | 20 |
| LC | 519 | C | A | 0.006803289 | 0.000549993 | 150 | 20 |
| LC | 521 | A | C | 0.0001102 | 0.001572387 | 150 | 20 |
| LC | 523 | C | A | 0.001119571 | 0.00102156 | 150 | 20 |
| LC | 533 | C | A | 0.003414364 | 0.00093978 | 150 | 20 |
| LC | 534 | C | A | 0.002222647 | 0.000716573 | 150 | 20 |
| LC | 543 | G | A | 0.000436308 | 0.000535583 | 150 | 20 |
| LC | 561 | C | A | 0.001222827 | 0.0005675 | 150 | 20 |
| LC | 565 | C | A | 0.000124777 | 0.000637097 | 150 | 20 |
| LC | 573 | C | A | 0.002421428 | 0.001006197 | 150 | 20 |
| LC | 576 | C | T | 0.000215287 | 0.000978843 | 150 | 20 |
| LC | 579 | C | T | 0.000104308 | 0.001168943 | 150 | 20 |
| LC | 582 | G | T | 0.008329098 | 0.001235637 | 150 | 20 |
| LC | 583 | G | T | 0.00281803 | 0.001691503 | 150 | 20 |
| LC | 586 | T | G | 0.008743975 | 0.001048437 | 150 | 20 |
| LC | 587 | C | T | 0.009387118 | 0.0006323 | 150 | 20 |
| LC | 590 | T | G | 0.004694043 | 0.00088384 | 150 | 20 |
| LC | 593 | C | T | 0.008789137 | 0.000613553 | 150 | 20 |
| LC | 595 | G | T | 0.001453916 | 0.000805487 | 150 | 20 |
| LC | 597 | G | T | 0.002642232 | 0.000642007 | 150 | 20 |
| LC | 600 | G | T | 0.000253398 | 0.00078928 | 150 | 20 |
| LC | 601 | G | T | 0.008489268 | 0.00106859 | 150 | 20 |
| LC | 720 | C | A | 0.008426751 | 0.0007572 | 150 | 20 |
| LC | 729 | C | A | 0.008202994 | 0.000593573 | 150 | 20 |
| LC | 731 | A | C | 0.000115895 | 0.000945383 | 150 | 20 |
| LC | 747 | A | C | 0.002586288 | 0.00098281 | 150 | 20 |
| LC | 148 | A | C | 0.001166798 | 0.00153342 | 150 | 0 |
| LC | 181 | A | C | 0.003017291 | 0.002526217 | 150 | 0 |
| LC | 183 | C | A | 0.000251702 | 0.00082396 | 150 | 0 |
| LC | 185 | C | A | 0.00812257 | 0.001136373 | 150 | 0 |
| LC | 186 | C | A | 0.00309411 | 0.00082827 | 150 | 0 |
| LC | 191 | A | C | 0.000661996 | 0.00172167 | 150 | 0 |
| LC | 193 | T | A | 0.006590619 | 0.000730763 | 150 | 0 |
| LC | 194 | C | A | 0.002054532 | 0.001909773 | 150 | 0 |
| LC | 206 | G | T | 4.64E-05 | 0.000834493 | 150 | 0 |
| LC | 208 | T | G | 0.000463972 | 0.00095827 | 150 | 0 |
| LC | 224 | G | T | 0.007140816 | 0.000870493 | 150 | 0 |
| LC | 236 | G | T | 0.009643968 | 0.000871397 | 150 | 0 |
| LC | 243 | G | T | 0.006316701 | 0.00074962 | 150 | 0 |
| LC | 252 | G | T | 0.002863926 | 0.001199973 | 150 | 0 |
| LC | 267 | G | T | 0.007241776 | 0.000586977 | 150 | 0 |
| LC | 271 | G | T | 0.004726517 | 0.00088723 | 150 | 0 |
| LC | 284 | G | T | 0.0006642 | 0.0007437 | 150 | 0 |
| LC | 298 | G | T | 0.003307987 | 0.000625277 | 150 | 0 |
| LC | 302 | G | T | 0.009411851 | 0.000657563 | 150 | 0 |
| LC | 326 | T | C | 0.001132147 | 0.000518333 | 150 | 0 |
| LC | 336 | G | T | 0.006259833 | 0.00084808 | 150 | 0 |
| LC | 343 | G | T | 0.000380699 | 0.00093869 | 150 | 0 |
| LC | 345 | G | T | 0.000934799 | 0.000862717 | 150 | 0 |
| LC | 353 | C | T | 0.003495725 | 0.000653027 | 150 | 0 |
| LC | 377 | G | T | 0.00262504 | 0.00087023 | 150 | 0 |
| LC | 378 | G | A | 0.000783941 | 0.006498197 | 150 | 0 |
| LC | 397 | G | T | 0.00034081 | 0.000634717 | 150 | 0 |
| LC | 398 | G | T | 0.009389865 | 0.000870147 | 150 | 0 |
| LC | 413 | T | G | 0.000844978 | 0.001779083 | 150 | 0 |
| LC | 415 | G | T | 0.002994472 | 0.000909533 | 150 | 0 |
| LC | 423 | G | T | 0.008458564 | 0.000716597 | 150 | 0 |
| LC | 429 | G | T | 0.000215209 | 0.000552597 | 150 | 0 |
| LC | 453 | C | A | 5.42E-05 | 0.00058478 | 150 | 0 |
| LC | 456 | C | A | 0.006215412 | 0.000558153 | 150 | 0 |
| LC | 483 | C | A | 0.006919881 | 0.000616487 | 150 | 0 |
| LC | 487 | A | C | 0.001623566 | 0.00077216 | 150 | 0 |
| LC | 491 | C | A | 0.000607071 | 0.000526713 | 150 | 0 |
| LC | 495 | C | A | 0.000386728 | 0.000726567 | 150 | 0 |
| LC | 515 | A | C | 0.000245959 | 0.000953853 | 150 | 0 |
| LC | 516 | C | A | 0.002242489 | 0.000674397 | 150 | 0 |
| LC | 523 | C | A | 2.29E-05 | 0.00054766 | 150 | 0 |
| LC | 526 | C | A | 0.00033126 | 0.000513717 | 150 | 0 |
| LC | 533 | C | A | 0.008427463 | 0.000820663 | 150 | 0 |
| LC | 534 | C | A | 0.002182804 | 0.001083483 | 150 | 0 |
| LC | 564 | G | A | 0.009689903 | 0.000521183 | 150 | 0 |
| LC | 565 | C | A | 0.007612571 | 0.000772807 | 150 | 0 |
| LC | 569 | C | A | 0.006653483 | 0.000785907 | 150 | 0 |
| LC | 573 | C | A | 0.000105803 | 0.00088174 | 150 | 0 |
| LC | 582 | G | T | 0.000515437 | 0.001194867 | 150 | 0 |
| LC | 595 | G | T | 0.00141577 | 0.000724383 | 150 | 0 |
| LC | 720 | C | A | 0.003705507 | 0.000672567 | 150 | 0 |
| LC | 726 | C | A | 0.005077965 | 0.00051495 | 150 | 0 |
| LC | 729 | C | A | 0.002578568 | 0.000781113 | 150 | 0 |
| LC | 747 | A | C | 0.000163372 | 0.001053183 | 150 | 0 |
| LC | 173 | G | A | 0.000598829 | 0.000543993 | 100 | 80 |
| LC | 179 | C | A | 2.74E-05 | 0.000986913 | 100 | 80 |
| LC | 181 | A | C | 0.009224566 | 0.00053564 | 100 | 80 |
| LC | 184 | T | A | 0.004662491 | 0.001028737 | 100 | 80 |
| LC | 189 | C | A | 0.007700254 | 0.000698333 | 100 | 80 |
| LC | 463 | T | A | 3.23E-05 | 0.01402813 | 100 | 80 |
| LC | 596 | A | T | 0.002035497 | 0.00083556 | 100 | 80 |
| LC | 123 | G | A | 0.004664869 | 0.00079385 | 100 | 20 |
| LC | 189 | C | A | 0.006310522 | 0.000539463 | 100 | 20 |
| LC | 198 | C | A | 0.006038856 | 0.00058081 | 100 | 20 |
| LC | 521 | A | C | 0.002436998 | 0.000994987 | 100 | 20 |
| LC | 596 | A | T | 0.001297359 | 0.000503993 | 100 | 20 |
| LC | 181 | A | C | 0.006251679 | 0.000643817 | 100 | 0 |
| LC | 521 | A | C | 0.00499697 | 0.00094679 | 100 | 0 |
| LC | 525 | T | A | 6.17E-05 | 0.000636133 | 100 | 0 |
| LC | 572 | G | A | 0.000745171 | 0.000717057 | 100 | 0 |
| LC | 332 | C | A | 0.008667923 | 0.000809853 | 50 | 80 |
| LC | 123 | G | A | 0.000911795 | 0.001282437 | 50 | 20 |
| LC | 128 | T | I | 0.008678203 | 0.000533147 | 50 | 20 |
| HC | 36 | T | G | 0.000165275 | 0.00092491 | 150 | 80 |
| HC | 40 | G | T | 0.002743816 | 0.000734793 | 150 | 80 |
| HC | 102 | A | T | 0.000259956 | 0.000851783 | 150 | 80 |
| HC | 104 | T | A | 0.001089288 | 0.0007058 | 150 | 80 |
| HC | 148 | C | A | 5.28E-05 | 0.000626037 | 150 | 80 |
| HC | 159 | C | A | 0.000203815 | 0.000764697 | 150 | 80 |
| HC | 160 | C | A | 0.006548216 | 0.000827183 | 150 | 80 |
| HC | 163 | C | A | 0.001945078 | 0.00087885 | 150 | 80 |
| HC | 165 | A | C | 0.001076864 | 0.00127299 | 150 | 80 |
| HC | 166 | C | A | 0.001403943 | 0.001112733 | 150 | 80 |
| HC | 167 | A | C | 0.000137071 | 0.001943873 | 150 | 80 |
| HC | 168 | C | A | 0.007615629 | 0.001620607 | 150 | 80 |
| HC | 169 | C | A | 0.007015346 | 0.001078633 | 150 | 80 |
| HC | 172 | C | A | 0.000175704 | 0.001211017 | 150 | 80 |
| HC | 173 | A | C | 0.001708858 | 0.001256377 | 150 | 80 |
| HC | 174 | C | A | 0.000153918 | 0.00177281 | 150 | 80 |
| HC | 175 | C | A | 0.008670018 | 0.00108855 | 150 | 80 |
| HC | 177 | A | C | 0.003145351 | 0.001173627 | 150 | 80 |
| HC | 178 | C | A | 9.75E-05 | 0.001263543 | 150 | 80 |
| HC | 180 | A | C | 0.004644043 | 0.00178913 | 150 | 80 |
| HC | 181 | C | A | 0.00181704 | 0.001282103 | 150 | 80 |
| HC | 183 | A | C | 0.001878229 | 0.002280827 | 150 | 80 |
| HC | 184 | C | A | 0.000287994 | 0.00145202 | 150 | 80 |
| HC | 189 | A | C | 0.002063999 | 0.001463463 | 150 | 80 |
| HC | 190 | C | A | 0.003115111 | 0.001464033 | 150 | 80 |
| HC | 201 | A | C | 0.005030379 | 0.000856827 | 150 | 80 |
| HC | 204 | C | A | 0.007473686 | 0.00168794 | 150 | 80 |
| HC | 206 | C | A | 0.004538287 | 0.003249063 | 150 | 80 |
| HC | 210 | G | T | 0.006307468 | 0.000740853 | 150 | 80 |
| HC | 214 | G | T | 0.001145094 | 0.000628363 | 150 | 80 |
| HC | 216 | G | T | 0.000843848 | 0.000959243 | 150 | 80 |
| HC | 221 | G | T | 0.000229421 | 0.000883 | 150 | 80 |
| HC | 224 | T | G | 0.009909632 | 0.00112194 | 150 | 80 |
| HC | 231 | G | T | 0.000719077 | 0.000634217 | 150 | 80 |
| HC | 235 | C | T | 0.006617889 | 0.00070327 | 150 | 80 |
| HC | 265 | C | T | 0.000853528 | 0.00057818 | 150 | 80 |
| HC | 286 | G | T | 0.000548407 | 0.001098013 | 150 | 80 |
| HC | 294 | T | C | 0.006388069 | 0.00065321 | 150 | 80 |
| HC | 300 | C | T | 0.001888388 | 0.000797997 | 150 | 80 |
| HC | 307 | C | T | 0.009575949 | 0.00054508 | 150 | 80 |
| HC | 329 | G | T | 0.001230084 | 0.000667433 | 150 | 80 |
| HC | 346 | G | T | 0.009992471 | 0.00054554 | 150 | 80 |
| HC | 378 | G | T | 0.000946116 | 0.000583103 | 150 | 80 |
| HC | 400 | G | T | 0.003768131 | 0.001022617 | 150 | 80 |
| HC | 401 | G | T | 0.00010469 | 0.00086655 | 150 | 80 |
| HC | 406 | C | T | 0.000859957 | 0.000540037 | 150 | 80 |
| HC | 419 | C | A | 0.004816263 | 0.000668247 | 150 | 80 |
| HC | 420 | A | T | 0.003928475 | 0.00130764 | 150 | 80 |
| HC | 423 | G | A | 0.002808788 | 0.000613623 | 150 | 80 |
| HC | 441 | C | A | 0.002971418 | 0.000606547 | 150 | 80 |
| HC | 452 | A | C | 0.000213376 | 0.00130499 | 150 | 80 |
| HC | 454 | C | A | 0.006211883 | 0.000644543 | 150 | 80 |
| HC | 460 | C | A | 0.002014217 | 0.000672137 | 150 | 80 |
| HC | 465 | C | A | 0.00163173 | 0.00093641 | 150 | 80 |
| HC | 476 | C | T | 0.00396123 | 0.004000163 | 150 | 80 |
| HC | 480 | C | A | 0.002386203 | 0.000699513 | 150 | 80 |
| HC | 481 | C | A | 0.00769984 | 0.00137233 | 150 | 80 |
| HC | 483 | C | A | 0.00825888 | 0.000828783 | 150 | 80 |
| HC | 521 | G | T | 0.00018495 | 0.001083813 | 150 | 80 |
| HC | 531 | T | G | 0.000411894 | 0.001761937 | 150 | 80 |
| HC | 536 | G | T | 0.002761919 | 0.000850727 | 150 | 80 |
| HC | 547 | T | C | 0.00185749 | 0.000538543 | 150 | 80 |
| HC | 562 | G | T | 0.003494666 | 0.000756123 | 150 | 80 |
| HC | 568 | G | T | 0.004252459 | 0.000963433 | 150 | 80 |
| HC | 590 | G | T | 0.006473926 | 0.000528573 | 150 | 80 |
| HC | 608 | G | T | 0.002434276 | 0.000934787 | 150 | 80 |
| HC | 642 | C | A | 0.007680474 | 0.00061288 | 150 | 80 |
| HC | 660 | C | A | 0.003931014 | 0.000875627 | 150 | 80 |
| HC | 663 | C | A | 0.001137753 | 0.00102122 | 150 | 80 |
| HC | 666 | C | A | 0.008171628 | 0.000585727 | 150 | 80 |
| HC | 667 | C | A | 0.007533501 | 0.000754687 | 150 | 80 |
| HC | 670 | C | A | 0.000276934 | 0.000646647 | 150 | 80 |
| HC | 677 | A | C | 0.005128258 | 0.001414393 | 150 | 80 |
| HC | 678 | C | A | 1.31E-06 | 0.000903713 | 150 | 80 |
| HC | 687 | A | C | 0.003868819 | 0.000745043 | 150 | 80 |
| HC | 689 | A | C | 0.008367202 | 0.001909623 | 150 | 80 |
| HC | 691 | C | A | 0.005579863 | 0.000652537 | 150 | 80 |
| HC | 694 | C | A | 0.001303022 | 0.000896033 | 150 | 80 |
| HC | 696 | A | C | 0.006177114 | 0.000755607 | 150 | 80 |
| HC | 704 | C | A | 0.00080508 | 0.00079722 | 150 | 80 |
| HC | 706 | C | A | 0.007444015 | 0.000967163 | 150 | 80 |
| HC | 710 | C | A | 0.004393155 | 0.00122192 | 150 | 80 |
| HC | 714 | C | A | 0.003749017 | 0.000909447 | 150 | 80 |
| HC | 718 | C | A | 0.005363875 | 0.000798907 | 150 | 80 |
| HC | 719 | A | C | 0.000813978 | 0.001979363 | 150 | 80 |
| HC | 721 | C | A | 0.005039556 | 0.00120448 | 150 | 80 |
| HC | 730 | C | A | 0.003739995 | 0.000883173 | 150 | 80 |
| HC | 744 | C | A | 1.47E-05 | 0.00091702 | 150 | 80 |
| HC | 750 | A | C | 0.001722573 | 0.001222047 | 150 | 80 |
| HC | 755 | C | A | 0.004286982 | 0.001035877 | 150 | 80 |
| HC | 758 | C | A | 0.002116232 | 0.000966437 | 150 | 80 |
| HC | 770 | T | G | 0.008180584 | 0.001179673 | 150 | 80 |
| HC | 791 | G | T | 0.006986048 | 0.00083429 | 150 | 80 |
| HC | 795 | G | T | 0.001280831 | 0.000882937 | 150 | 80 |
| HC | 805 | G | T | 0.004123795 | 0.000876677 | 150 | 80 |
| HC | 819 | C | D | 0.005812735 | 0.00067804 | 150 | 80 |
| HC | 823 | G | T | 0.009315698 | 0.00074975 | 150 | 80 |
| HC | 838 | G | T | 0.003935764 | 0.00064412 | 150 | 80 |
| HC | 861 | T | G | 0.004463947 | 0.00102297 | 150 | 80 |
| HC | 862 | G | T | 0.008806789 | 0.000567153 | 150 | 80 |
| HC | 871 | G | T | 0.002156002 | 0.000523907 | 150 | 80 |
| HC | 873 | T | G | 0.003656733 | 0.001170803 | 150 | 80 |
| HC | 878 | G | T | 0.004630207 | 0.000553243 | 150 | 80 |
| HC | 889 | G | T | 0.000887661 | 0.000768677 | 150 | 80 |
| HC | 890 | G | T | 0.008428554 | 0.000924397 | 150 | 80 |
| HC | 899 | G | T | 0.005286964 | 0.000746953 | 150 | 80 |
| HC | 901 | G | T | 0.007166924 | 0.000757207 | 150 | 80 |
| HC | 902 | G | T | 0.007726263 | 0.000749433 | 150 | 80 |
| HC | 903 | T | G | 2.83E-05 | 0.000814917 | 150 | 80 |
| HC | 916 | G | T | 0.001900402 | 0.0009558 | 150 | 80 |
| HC | 917 | T | G | 0.003150875 | 0.001368117 | 150 | 80 |
| HC | 923 | G | T | 0.003076632 | 0.000830717 | 150 | 80 |
| HC | 931 | G | T | 0.007535534 | 0.00058965 | 150 | 80 |
| HC | 932 | G | T | 0.00389223 | 0.00063244 | 150 | 80 |
| HC | 956 | C | A | 0.000755826 | 0.00091746 | 150 | 80 |
| HC | 957 | C | A | 0.004486099 | 0.000773717 | 150 | 80 |
| HC | 959 | C | A | 0.007213236 | 0.00070847 | 150 | 80 |
| HC | 980 | A | G | 0.009885299 | 0.001144733 | 150 | 80 |
| HC | 982 | C | A | 0.000333731 | 0.000584897 | 150 | 80 |
| HC | 986 | C | A | 0.000396238 | 0.00072225 | 150 | 80 |
| HC | 996 | C | A | 0.002275797 | 0.000686003 | 150 | 80 |
| HC | 1020 | A | C | 0.00524001 | 0.00119978 | 150 | 80 |
| HC | 1057 | C | A | 9.14E-05 | 0.00065032 | 150 | 80 |
| HC | 1060 | C | A | 0.000484845 | 0.001028983 | 150 | 80 |
| HC | 1066 | C | A | 0.003991183 | 0.00079268 | 150 | 80 |
| HC | 1070 | C | G | 0.00026733 | 0.01032192 | 150 | 80 |
| HC | 1072 | C | A | 0.003871428 | 0.00062735 | 150 | 80 |
| HC | 1074 | C | A | 0.006554116 | 0.000934903 | 150 | 80 |
| HC | 1077 | C | A | 0.000562559 | 0.000845057 | 150 | 80 |
| HC | 1078 | C | A | 0.005938624 | 0.001201033 | 150 | 80 |
| HC | 1090 | C | A | 0.008602016 | 0.000843873 | 150 | 80 |
| HC | 1093 | C | A | 0.002212281 | 0.000807447 | 150 | 80 |
| HC | 1095 | C | A | 0.004536837 | 0.00121179 | 150 | 80 |
| HC | 1096 | C | A | 0.006620307 | 0.000720593 | 150 | 80 |
| HC | 1119 | A | C | 0.002385689 | 0.000558353 | 150 | 80 |
| HC | 1143 | C | A | 0.005344573 | 0.000775383 | 150 | 80 |
| HC | 1148 | C | A | 0.00023906 | 0.000863673 | 150 | 80 |
| HC | 1157 | A | C | 0.000771158 | 0.000567237 | 150 | 80 |
| HC | 1171 | G | T | 0.007231092 | 0.000539973 | 150 | 80 |
| HC | 1176 | C | A | 0.00996369 | 0.000692263 | 150 | 80 |
| HC | 1177 | C | A | 0.000454799 | 0.000597233 | 150 | 80 |
| HC | 1178 | C | A | 0.001334311 | 0.000728767 | 150 | 80 |
| HC | 1187 | C | A | 0.009080281 | 0.00072284 | 150 | 80 |
| HC | 1209 | C | A | 0.009531219 | 0.000765457 | 150 | 80 |
| HC | 1234 | C | A | 0.002392215 | 0.00104525 | 150 | 80 |
| HC | 1239 | G | A | 0.000147619 | 0.000872707 | 150 | 80 |
| HC | 1240 | C | A | 0.003141813 | 0.00107449 | 150 | 80 |
| HC | 1241 | C | A | 0.002468135 | 0.001048597 | 150 | 80 |
| HC | 1246 | A | G | 0.000674207 | 0.00737989 | 150 | 80 |
| HC | 1252 | C | A | 0.002176399 | 0.000980077 | 150 | 80 |
| HC | 1254 | A | C | 0.001876254 | 0.000897993 | 150 | 80 |
| HC | 1255 | C | A | 0.001126756 | 0.000920267 | 150 | 80 |
| HC | 1264 | C | T | 0.004270523 | 0.000605393 | 150 | 80 |
| HC | 1267 | C | T | 0.003319084 | 0.001038317 | 150 | 80 |
| HC | 1274 | G | T | 0.000466841 | 0.001691943 | 150 | 80 |
| HC | 1275 | T | G | 0.006076298 | 0.002104493 | 150 | 80 |
| HC | 1276 | G | T | 0.007207001 | 0.001388497 | 150 | 80 |
| HC | 1277 | C | T | 0.004974363 | 0.000574737 | 150 | 80 |
| HC | 1279 | G | T | 0.001663101 | 0.001384073 | 150 | 80 |
| HC | 1280 | G | T | 7.54E-05 | 0.00187718 | 150 | 80 |
| HC | 1283 | T | G | 0.001910728 | 0.000797243 | 150 | 80 |
| HC | 1286 | G | T | 0.007313138 | 0.001438923 | 150 | 80 |
| HC | 1289 | G | T | 1.75E-06 | 0.00087923 | 150 | 80 |
| HC | 1290 | G | T | 0.002559643 | 0.00092213 | 150 | 80 |
| HC | 1298 | T | G | 0.006217323 | 0.000733693 | 150 | 80 |
| HC | 1303 | G | T | 0.004752984 | 0.001054217 | 150 | 80 |
| HC | 1304 | T | G | 1.17E-06 | 0.001307023 | 150 | 80 |
| HC | 1311 | G | T | 0.000432847 | 0.000510777 | 150 | 80 |
| HC | 1392 | A | C | 0.000951186 | 0.000644337 | 150 | 80 |
| HC | 1394 | A | G | 0.007207499 | 0.001532903 | 150 | 80 |
| HC | 1401 | A | C | 0.00132787 | 0.000547847 | 150 | 80 |
| HC | 1418 | C | A | 0.007256309 | 0.00107686 | 150 | 80 |
| HC | 1423 | C | A | 0.007624086 | 0.00132203 | 150 | 80 |
| HC | 36 | T | G | 0.001648575 | 0.00074171 | 150 | 20 |
| HC | 40 | G | T | 0.000758382 | 0.000627663 | 150 | 20 |
| HC | 156 | C | A | 0.003604421 | 0.000571573 | 150 | 20 |
| HC | 159 | C | A | 0.000392777 | 0.000684563 | 150 | 20 |
| HC | 163 | C | A | 7.73E-05 | 0.000935617 | 150 | 20 |
| HC | 165 | A | C | 5.28E-08 | 0.00109607 | 150 | 20 |
| HC | 167 | A | C | 0.001808261 | 0.0017914 | 150 | 20 |
| HC | 168 | C | A | 0.003899961 | 0.001467093 | 150 | 20 |
| HC | 169 | C | A | 7.63E-05 | 0.00090111 | 150 | 20 |
| HC | 172 | C | A | 0.003836827 | 0.00109428 | 150 | 20 |
| HC | 173 | A | C | 0.009757979 | 0.001023557 | 150 | 20 |
| HC | 174 | C | A | 1.64E-06 | 0.001627407 | 150 | 20 |
| HC | 175 | C | A | 0.001508342 | 0.00144433 | 150 | 20 |
| HC | 177 | A | C | 0.001615749 | 0.00108925 | 150 | 20 |
| HC | 178 | C | A | 0.000128948 | 0.00123672 | 150 | 20 |
| HC | 180 | A | C | 0.007019123 | 0.001758693 | 150 | 20 |
| HC | 181 | C | A | 0.005772424 | 0.001250147 | 150 | 20 |
| HC | 183 | A | C | 0.004047628 | 0.001679233 | 150 | 20 |
| HC | 184 | C | A | 0.007837524 | 0.001500917 | 150 | 20 |
| HC | 186 | T | A | 0.002567879 | 0.000786043 | 150 | 20 |
| HC | 189 | A | C | 0.001119134 | 0.001443713 | 150 | 20 |
| HC | 194 | G | A | 7.86E-06 | 0.00119 | 150 | 20 |
| HC | 196 | G | A | 0.002469773 | 0.0010378 | 150 | 20 |
| HC | 200 | C | A | 0.005759384 | 0.000834137 | 150 | 20 |
| HC | 204 | C | A | 0.000639908 | 0.00199651 | 150 | 20 |
| HC | 205 | C | A | 0.008158958 | 0.003087837 | 150 | 20 |
| HC | 206 | C | A | 0.000697035 | 0.003585103 | 150 | 20 |
| HC | 221 | G | T | 0.001210054 | 0.000772023 | 150 | 20 |
| HC | 224 | T | G | 0.006641289 | 0.001028597 | 150 | 20 |
| HC | 225 | G | T | 0.000271903 | 0.00073828 | 150 | 20 |
| HC | 230 | G | T | 0.006142853 | 0.001183787 | 150 | 20 |
| HC | 234 | G | A | 0.003428035 | 0.000800307 | 150 | 20 |
| HC | 265 | C | T | 5.44E-05 | 0.000966323 | 150 | 20 |
| HC | 275 | T | G | 0.001977005 | 0.001288143 | 150 | 20 |
| HC | 294 | T | C | 0.00701111 | 0.000733113 | 150 | 20 |
| HC | 328 | G | T | 0.002232226 | 0.0007884 | 150 | 20 |
| HC | 329 | G | T | 0.003642906 | 0.000535547 | 150 | 20 |
| HC | 338 | T | G | 0.0093911 | 0.001085737 | 150 | 20 |
| HC | 378 | G | T | 0.002528688 | 0.000646743 | 150 | 20 |
| HC | 395 | G | T | 0.002398977 | 0.000617093 | 150 | 20 |
| HC | 401 | G | T | 0.001624294 | 0.000886803 | 150 | 20 |
| HC | 414 | G | T | 0.00833889 | 0.000830407 | 150 | 20 |
| HC | 420 | A | T | 0.009693603 | 0.00238159 | 150 | 20 |
| HC | 434 | A | C | 0.000159211 | 0.00108614 | 150 | 20 |
| HC | 437 | G | A | 0.006828808 | 0.000569303 | 150 | 20 |
| HC | 441 | C | A | 0.002789012 | 0.000817793 | 150 | 20 |
| HC | 446 | G | A | 0.009240356 | 0.00077135 | 150 | 20 |
| HC | 448 | T | C | 0.009570092 | 0.00052483 | 150 | 20 |
| HC | 452 | A | C | 0.007565637 | 0.001593587 | 150 | 20 |
| HC | 460 | C | A | 0.005774227 | 0.00061271 | 150 | 20 |
| HC | 461 | C | A | 0.008890072 | 0.000739267 | 150 | 20 |
| HC | 462 | C | A | 0.000565829 | 0.000541433 | 150 | 20 |
| HC | 465 | C | A | 0.002352408 | 0.0006139 | 150 | 20 |
| HC | 483 | C | A | 0.000175945 | 0.000513913 | 150 | 20 |
| HC | 503 | G | T | 5.49E-05 | 0.00078656 | 150 | 20 |
| HC | 520 | G | T | 0.002048583 | 0.000736023 | 150 | 20 |
| HC | 525 | G | T | 0.001076982 | 0.000991407 | 150 | 20 |
| HC | 532 | G | T | 0.003341551 | 0.000855807 | 150 | 20 |
| HC | 536 | G | T | 0.001348497 | 0.00090587 | 150 | 20 |
| HC | 548 | G | T | 0.007854201 | 0.00075244 | 150 | 20 |
| HC | 551 | C | T | 0.001126695 | 0.00056845 | 150 | 20 |
| HC | 567 | G | T | 0.006599362 | 0.00059717 | 150 | 20 |
| HC | 568 | G | T | 0.000387702 | 0.000729503 | 150 | 20 |
| HC | 628 | C | A | 0.006635158 | 0.000579027 | 150 | 20 |
| HC | 638 | C | A | 0.007685341 | 0.00052884 | 150 | 20 |
| HC | 643 | C | A | 0.004174456 | 0.000695787 | 150 | 20 |
| HC | 645 | C | A | 0.003339978 | 0.00052762 | 150 | 20 |
| HC | 667 | C | A | 0.001213072 | 0.00052214 | 150 | 20 |
| HC | 670 | C | A | 0.006219518 | 0.000816433 | 150 | 20 |
| HC | 676 | C | A | 2.24E-05 | 0.0006324 | 150 | 20 |
| HC | 677 | A | C | 0.008201465 | 0.001301547 | 150 | 20 |
| HC | 679 | C | A | 0.002184145 | 0.00084349 | 150 | 20 |
| HC | 685 | C | A | 3.24E-05 | 0.0005931 | 150 | 20 |
| HC | 694 | C | A | 0.00450537 | 0.000508247 | 150 | 20 |
| HC | 696 | A | C | 0.000453365 | 0.00061588 | 150 | 20 |
| HC | 710 | C | A | 0.000581944 | 0.000906527 | 150 | 20 |
| HC | 719 | A | C | 0.001285959 | 0.001529563 | 150 | 20 |
| HC | 721 | C | A | 7.47E-05 | 0.000885707 | 150 | 20 |
| HC | 745 | C | A | 0.001592187 | 0.0008991 | 150 | 20 |
| HC | 754 | C | A | 0.004638865 | 0.000991057 | 150 | 20 |
| HC | 755 | C | A | 0.00668213 | 0.00092341 | 150 | 20 |
| HC | 758 | C | A | 0.000864668 | 0.000798777 | 150 | 20 |
| HC | 770 | T | G | 0.000216442 | 0.000916073 | 150 | 20 |
| HC | 791 | G | T | 2.97E-05 | 0.000710577 | 150 | 20 |
| HC | 795 | G | T | 7.63E-05 | 0.000761467 | 150 | 20 |
| HC | 806 | T | G | 0.00733719 | 0.000562553 | 150 | 20 |
| HC | 838 | G | T | 0.006905113 | 0.000604913 | 150 | 20 |
| HC | 841 | G | T | 0.000590267 | 0.000566987 | 150 | 20 |
| HC | 849 | G | T | 0.000928417 | 0.00088903 | 150 | 20 |
| HC | 861 | T | G | 0.003927713 | 0.000986267 | 150 | 20 |
| HC | 874 | G | T | 0.000262747 | 0.000673787 | 150 | 20 |
| HC | 877 | G | T | 0.000336541 | 0.00054327 | 150 | 20 |
| HC | 895 | T | G | 0.00502015 | 0.00061858 | 150 | 20 |
| HC | 901 | G | T | 0.00471692 | 0.000669437 | 150 | 20 |
| HC | 902 | G | T | 0.001351227 | 0.000756577 | 150 | 20 |
| HC | 903 | T | G | 0.00842927 | 0.00107032 | 150 | 20 |
| HC | 908 | T | G | 0.002082231 | 0.00098591 | 150 | 20 |
| HC | 916 | G | T | 0.006010085 | 0.001072147 | 150 | 20 |
| HC | 917 | T | G | 0.0076791 | 0.001415447 | 150 | 20 |
| HC | 922 | G | T | 2.56E-05 | 0.000732847 | 150 | 20 |
| HC | 923 | G | T | 0.006664569 | 0.000703987 | 150 | 20 |
| HC | 926 | G | T | 0.001652232 | 0.000721913 | 150 | 20 |
| HC | 932 | G | T | 0.002134308 | 0.00058191 | 150 | 20 |
| HC | 937 | G | A | 0.00348738 | 0.000683007 | 150 | 20 |
| HC | 955 | G | T | 0.008234035 | 0.000523837 | 150 | 20 |
| HC | 956 | C | A | 0.005075581 | 0.000834507 | 150 | 20 |
| HC | 959 | C | A | 0.004211653 | 0.000773277 | 150 | 20 |
| HC | 965 | G | A | 0.00149037 | 0.000638527 | 150 | 20 |
| HC | 980 | A | G | 0.003308314 | 0.00099172 | 150 | 20 |
| HC | 982 | C | A | 0.009662652 | 0.00052179 | 150 | 20 |
| HC | 986 | C | A | 0.000755202 | 0.000796353 | 150 | 20 |
| HC | 996 | C | A | 0.000641668 | 0.00058483 | 150 | 20 |
| HC | 1014 | A | G | 0.007192033 | 0.000523577 | 150 | 20 |
| HC | 1021 | C | A | 5.07E-07 | 0.00080312 | 150 | 20 |
| HC | 1032 | G | A | 0.002213438 | 0.00053362 | 150 | 20 |
| HC | 1057 | C | A | 0.008559594 | 0.000823223 | 150 | 20 |
| HC | 1067 | C | A | 4.24E-05 | 0.000854267 | 150 | 20 |
| HC | 1072 | C | A | 0.000475778 | 0.000610737 | 150 | 20 |
| HC | 1074 | C | A | 0.009533333 | 0.00091576 | 150 | 20 |
| HC | 1075 | C | A | 0.005117931 | 0.000522603 | 150 | 20 |
| HC | 1078 | C | A | 0.004588415 | 0.000808073 | 150 | 20 |
| HC | 1087 | A | T | 0.00565237 | 0.000547973 | 150 | 20 |
| HC | 1089 | C | A | 0.008245116 | 0.00103978 | 150 | 20 |
| HC | 1096 | C | A | 0.002346457 | 0.000935373 | 150 | 20 |
| HC | 1119 | A | C | 0.009610752 | 0.000563513 | 150 | 20 |
| HC | 1135 | C | A | 0.005190616 | 0.000642503 | 150 | 20 |
| HC | 1136 | C | A | 0.002662249 | 0.0008032 | 150 | 20 |
| HC | 1140 | C | A | 0.003063459 | 0.000582123 | 150 | 20 |
| HC | 1142 | C | A | 0.001405431 | 0.000945713 | 150 | 20 |
| HC | 1157 | A | C | 0.001931308 | 0.000657673 | 150 | 20 |
| HC | 1166 | A | C | 0.000920898 | 0.000525807 | 150 | 20 |
| HC | 1169 | C | A | 0.005153515 | 0.000658487 | 150 | 20 |
| HC | 1176 | C | A | 4.67E-05 | 0.000954923 | 150 | 20 |
| HC | 1178 | C | A | 0.000637691 | 0.000936173 | 150 | 20 |
| HC | 1187 | C | A | 0.001524023 | 0.000896153 | 150 | 20 |
| HC | 1198 | C | A | 0.00423126 | 0.000823503 | 150 | 20 |
| HC | 1203 | A | C | 0.00195684 | 0.001465647 | 150 | 20 |
| HC | 1204 | C | A | 0.00622097 | 0.000601853 | 150 | 20 |
| HC | 1205 | C | A | 0.000373841 | 0.000527517 | 150 | 20 |
| HC | 1210 | C | A | 0.000504137 | 0.000680703 | 150 | 20 |
| HC | 1239 | G | A | 0.002333844 | 0.000511923 | 150 | 20 |
| HC | 1240 | C | A | 0.003185709 | 0.00088142 | 150 | 20 |
| HC | 1241 | C | A | 0.004073399 | 0.000918883 | 150 | 20 |
| HC | 1245 | C | A | 0.005566351 | 0.001270237 | 150 | 20 |
| HC | 1251 | A | C | 0.005667803 | 0.00077726 | 150 | 20 |
| HC | 1254 | A | C | 0.0025694 | 0.00101485 | 150 | 20 |
| HC | 1258 | C | T | 0.001673296 | 0.000746967 | 150 | 20 |
| HC | 1261 | G | T | 0.003325922 | 0.001476443 | 150 | 20 |
| HC | 1272 | C | T | 0.006309301 | 0.000553817 | 150 | 20 |
| HC | 1274 | G | T | 0.002638789 | 0.001449427 | 150 | 20 |
| HC | 1276 | G | T | 0.005806181 | 0.001276713 | 150 | 20 |
| HC | 1279 | G | T | 0.003023104 | 0.001256687 | 150 | 20 |
| HC | 1280 | G | T | 0.006797571 | 0.001650187 | 150 | 20 |
| HC | 1283 | T | G | 0.007510013 | 0.00072135 | 150 | 20 |
| HC | 1286 | G | T | 0.00219565 | 0.00107543 | 150 | 20 |
| HC | 1289 | G | T | 0.000394028 | 0.000780323 | 150 | 20 |
| HC | 1290 | G | T | 0.005822199 | 0.000648293 | 150 | 20 |
| HC | 1295 | T | G | 0.001574718 | 0.000527087 | 150 | 20 |
| HC | 1298 | T | G | 0.003620796 | 0.000557657 | 150 | 20 |
| HC | 1303 | G | T | 3.10E-07 | 0.000908163 | 150 | 20 |
| HC | 1304 | T | G | 0.002477647 | 0.001076587 | 150 | 20 |
| HC | 1394 | A | G | 0.00098495 | 0.001707237 | 150 | 20 |
| HC | 1405 | C | A | 2.15E-06 | 0.000742977 | 150 | 20 |
| HC | 1411 | C | A | 0.000486677 | 0.00056001 | 150 | 20 |
| HC | 1412 | C | A | 0.007801832 | 0.00059347 | 150 | 20 |
| HC | 1418 | C | A | 0.007637931 | 0.001108683 | 150 | 20 |
| HC | 36 | T | G | 0.000332358 | 0.000966793 | 150 | 0 |
| HC | 40 | G | T | 3.26E-05 | 0.00074945 | 150 | 0 |
| HC | 50 | T | G | 0.005140441 | 0.00070773 | 150 | 0 |
| HC | 165 | A | C | 0.003303277 | 0.001308077 | 150 | 0 |
| HC | 166 | C | A | 0.005958815 | 0.001128203 | 150 | 0 |
| HC | 167 | A | C | 0.000398421 | 0.001830317 | 150 | 0 |
| HC | 169 | C | A | 0.000714435 | 0.00130129 | 150 | 0 |
| HC | 172 | C | A | 0.001041938 | 0.001199833 | 150 | 0 |
| HC | 173 | A | C | 0.000257122 | 0.001064807 | 150 | 0 |
| HC | 174 | C | A | 0.003584754 | 0.001613107 | 150 | 0 |
| HC | 177 | A | C | 0.001994556 | 0.001162413 | 150 | 0 |
| HC | 178 | C | A | 0.000135253 | 0.001612693 | 150 | 0 |
| HC | 180 | A | C | 0.000393174 | 0.001686277 | 150 | 0 |
| HC | 181 | C | A | 0.001431181 | 0.001234383 | 150 | 0 |
| HC | 183 | A | C | 0.005532884 | 0.002077913 | 150 | 0 |
| HC | 184 | C | A | 3.03E-05 | 0.00146766 | 150 | 0 |
| HC | 185 | A | C | 2.19E-05 | 0.000976603 | 150 | 0 |
| HC | 193 | G | A | 0.007484773 | 0.001025863 | 150 | 0 |
| HC | 205 | C | A | 0.003105892 | 0.0023772 | 150 | 0 |
| HC | 216 | G | T | 1.94E-05 | 0.00074144 | 150 | 0 |
| HC | 224 | T | G | 0.002378391 | 0.00126516 | 150 | 0 |
| HC | 229 | G | T | 0.002574125 | 0.000775667 | 150 | 0 |
| HC | 230 | G | T | 0.003711377 | 0.000930843 | 150 | 0 |
| HC | 262 | C | T | 0.00451841 | 0.00050749 | 150 | 0 |
| HC | 289 | G | T | 0.003820152 | 0.000713027 | 150 | 0 |
| HC | 392 | T | G | 0.004546398 | 0.00093172 | 150 | 0 |
| HC | 414 | G | T | 6.47E-05 | 0.00054781 | 150 | 0 |
| HC | 454 | C | A | 0.004347662 | 0.000771347 | 150 | 0 |
| HC | 466 | C | A | 0.000781446 | 0.000606533 | 150 | 0 |
| HC | 472 | C | A | 0.004772487 | 0.000876257 | 150 | 0 |
| HC | 481 | C | A | 0.002162786 | 0.001087077 | 150 | 0 |
| HC | 482 | C | A | 0.007380641 | 0.00091515 | 150 | 0 |
| HC | 531 | T | G | 0.000564461 | 0.001710133 | 150 | 0 |
| HC | 535 | G | T | 0.000642471 | 0.000747007 | 150 | 0 |
| HC | 536 | G | T | 0.008583334 | 0.000943293 | 150 | 0 |
| HC | 562 | G | T | 0.002786215 | 0.00069248 | 150 | 0 |
| HC | 564 | G | T | 0.000210935 | 0.00059924 | 150 | 0 |
| HC | 568 | G | T | 0.003686463 | 0.000687727 | 150 | 0 |
| HC | 590 | G | T | 0.001209409 | 0.000501663 | 150 | 0 |
| HC | 637 | C | A | 0.001967303 | 0.00064705 | 150 | 0 |
| HC | 638 | C | A | 0.002842781 | 0.000735853 | 150 | 0 |
| HC | 645 | C | A | 0.00019533 | 0.000603917 | 150 | 0 |
| HC | 659 | C | A | 0.000511727 | 0.000709427 | 150 | 0 |
| HC | 670 | C | A | 0.000607873 | 0.000516413 | 150 | 0 |
| HC | 671 | C | A | 0.006382521 | 0.000531207 | 150 | 0 |
| HC | 677 | A | C | 0.001344621 | 0.00165178 | 150 | 0 |
| HC | 683 | A | C | 0.000814725 | 0.00108773 | 150 | 0 |
| HC | 685 | C | A | 0.007762969 | 0.00056965 | 150 | 0 |
| HC | 687 | A | C | 0.001160122 | 0.000823453 | 150 | 0 |
| HC | 690 | C | A | 0.004693764 | 0.000747567 | 150 | 0 |
| HC | 694 | C | A | 0.009652946 | 0.000842743 | 150 | 0 |
| HC | 704 | C | A | 0.006371815 | 0.000612297 | 150 | 0 |
| HC | 706 | C | A | 6.37E-07 | 0.00127799 | 150 | 0 |
| HC | 710 | C | A | 0.004178515 | 0.000795513 | 150 | 0 |
| HC | 714 | C | A | 0.001279433 | 0.001133093 | 150 | 0 |
| HC | 715 | C | A | 0.001121225 | 0.00097224 | 150 | 0 |
| HC | 720 | C | A | 0.006858796 | 0.000981093 | 150 | 0 |
| HC | 745 | C | A | 0.008704716 | 0.000908387 | 150 | 0 |
| HC | 750 | A | C | 0.000769182 | 0.00110257 | 150 | 0 |
| HC | 770 | T | G | 0.001433888 | 0.000843593 | 150 | 0 |
| HC | 791 | G | T | 0.005409849 | 0.001012437 | 150 | 0 |
| HC | 795 | G | T | 0.004249241 | 0.000737727 | 150 | 0 |
| HC | 805 | G | T | 0.006880164 | 0.00082001 | 150 | 0 |
| HC | 806 | T | G | 0.004339808 | 0.00089373 | 150 | 0 |
| HC | 812 | T | G | 0.007011805 | 0.000704713 | 150 | 0 |
| HC | 813 | T | G | 0.001949422 | 0.00059708 | 150 | 0 |
| HC | 830 | G | T | 0.007501185 | 0.000673777 | 150 | 0 |
| HC | 841 | G | T | 0.000118362 | 0.00070084 | 150 | 0 |
| HC | 849 | G | T | 0.000662119 | 0.00051918 | 150 | 0 |
| HC | 850 | G | T | 0.002998563 | 0.000597637 | 150 | 0 |
| HC | 859 | G | T | 0.001736444 | 0.000946383 | 150 | 0 |
| HC | 860 | G | T | 0.004462989 | 0.000835027 | 150 | 0 |
| HC | 873 | T | G | 0.000618916 | 0.001048717 | 150 | 0 |
| HC | 889 | G | T | 0.006703414 | 0.00067022 | 150 | 0 |
| HC | 893 | G | T | 0.006643785 | 0.000664863 | 150 | 0 |
| HC | 895 | T | G | 0.001994179 | 0.00067925 | 150 | 0 |
| HC | 901 | G | T | 0.008941245 | 0.000727533 | 150 | 0 |
| HC | 903 | T | G | 0.008484918 | 0.001095793 | 150 | 0 |
| HC | 915 | G | T | 0.002747458 | 0.00088157 | 150 | 0 |
| HC | 917 | T | G | 0.001240295 | 0.00154814 | 150 | 0 |
| HC | 926 | G | T | 0.007516036 | 0.00090106 | 150 | 0 |
| HC | 931 | G | T | 0.000175544 | 0.000531297 | 150 | 0 |
| HC | 934 | G | T | 0.004622531 | 0.000553683 | 150 | 0 |
| HC | 956 | C | A | 0.006886644 | 0.001080413 | 150 | 0 |
| HC | 984 | A | C | 0.009326551 | 0.001106783 | 150 | 0 |
| HC | 1020 | A | C | 3.03E-07 | 0.001168333 | 150 | 0 |
| HC | 1021 | C | A | 0.006289708 | 0.000849093 | 150 | 0 |
| HC | 1033 | C | A | 0.003989984 | 0.00063767 | 150 | 0 |
| HC | 1056 | G | A | 0.00147522 | 0.000711367 | 150 | 0 |
| HC | 1057 | C | A | 0.006540458 | 0.000819647 | 150 | 0 |
| HC | 1066 | C | A | 0.001528022 | 0.001089763 | 150 | 0 |
| HC | 1074 | C | A | 0.001478497 | 0.0006499 | 150 | 0 |
| HC | 1077 | C | A | 0.008200372 | 0.00090976 | 150 | 0 |
| HC | 1089 | C | A | 0.004228009 | 0.00078252 | 150 | 0 |
| HC | 1095 | C | A | 0.005219061 | 0.00108655 | 150 | 0 |
| HC | 1101 | C | A | 0.002476364 | 0.000889713 | 150 | 0 |
| HC | 1111 | G | T | 0.008513371 | 0.000735193 | 150 | 0 |
| HC | 1139 | C | A | 0.000302633 | 0.000825907 | 150 | 0 |
| HC | 1143 | C | A | 0.007200622 | 0.000757713 | 150 | 0 |
| HC | 1146 | G | A | 0.000553372 | 0.00053864 | 150 | 0 |
| HC | 1147 | C | A | 0.006755226 | 0.000905713 | 150 | 0 |
| HC | 1157 | A | C | 0.000454655 | 0.00070913 | 150 | 0 |
| HC | 1169 | C | A | 0.008015191 | 0.000703453 | 150 | 0 |
| HC | 1176 | C | A | 0.005024516 | 0.000704267 | 150 | 0 |
| HC | 1178 | C | A | 0.009210086 | 0.00065182 | 150 | 0 |
| HC | 1187 | C | A | 0.006289164 | 0.000616647 | 150 | 0 |
| HC | 1204 | C | A | 0.007254326 | 0.000786817 | 150 | 0 |
| HC | 1205 | C | A | 0.004965257 | 0.00071516 | 150 | 0 |
| HC | 1206 | C | A | 0.009295471 | 0.000806267 | 150 | 0 |
| HC | 1209 | C | A | 0.008039254 | 0.000734817 | 150 | 0 |
| HC | 1240 | C | A | 0.00053164 | 0.001120077 | 150 | 0 |
| HC | 1254 | A | C | 0.000621468 | 0.001048007 | 150 | 0 |
| HC | 1258 | C | T | 0.003746591 | 0.00082726 | 150 | 0 |
| HC | 1262 | A | T | 1.39E-05 | 0.000995543 | 150 | 0 |
| HC | 1274 | G | T | 0.002773426 | 0.001552763 | 150 | 0 |
| HC | 1275 | T | G | 0.002170289 | 0.001776787 | 150 | 0 |
| HC | 1277 | C | T | 0.005319094 | 0.000746293 | 150 | 0 |
| HC | 1279 | G | T | 0.00014895 | 0.00128893 | 150 | 0 |
| HC | 1280 | G | T | 0.005975504 | 0.001764987 | 150 | 0 |
| HC | 1283 | T | G | 0.001705974 | 0.000687893 | 150 | 0 |
| HC | 1286 | G | T | 0.006608941 | 0.001178977 | 150 | 0 |
| HC | 1298 | T | G | 0.004295472 | 0.000642983 | 150 | 0 |
| HC | 1304 | T | G | 0.009475016 | 0.001175477 | 150 | 0 |
| HC | 1330 | C | A | 0.001245216 | 0.009478873 | 150 | 0 |
| HC | 1372 | C | A | 0.000705154 | 0.009692363 | 150 | 0 |
| HC | 1392 | A | C | 0.008214122 | 0.000716963 | 150 | 0 |
| HC | 1394 | A | G | 8.39E-05 | 0.001732343 | 150 | 0 |
| HC | 1401 | A | C | 0.007791549 | 0.000523313 | 150 | 0 |
| HC | 1411 | C | A | 0.001285769 | 0.000659623 | 150 | 0 |
| HC | 1412 | C | A | 0.006427911 | 0.000653747 | 150 | 0 |
| HC | 1418 | C | A | 0.004774083 | 0.001131057 | 150 | 0 |
| HC | 1423 | C | A | 0.009856532 | 0.001563117 | 150 | 0 |
| HC | 182 | T | A | 0.003787935 | 0.000587333 | 100 | 80 |
| HC | 476 | C | T | 0.000638435 | 0.00150849 | 100 | 80 |
| HC | 725 | G | C | 0.004492056 | 0.000876843 | 100 | 80 |
| HC | 820 | T | C | 0.001993202 | 0.001674117 | 100 | 80 |
| HC | 1053 | T | C | 8.45E-05 | 0.000602447 | 100 | 80 |
| HC | 1070 | C | G | 0.001904384 | 0.001807833 | 100 | 80 |
| HC | 1246 | A | G | 0.000309033 | 0.008378363 | 100 | 80 |
| HC | 1262 | A | T | 0.000144746 | 0.000509503 | 100 | 80 |
| HC | 1264 | C | T | 0.000283325 | 0.000555293 | 100 | 80 |
| HC | 1280 | G | T | 3.58E-05 | 0.000869233 | 100 | 80 |
| HC | 1287 | A | T | 0.000859745 | 0.000504563 | 100 | 80 |
| HC | 1003 | G | T | 0.003342547 | 0.002223853 | 100 | 20 |
| HC | 1261 | G | T | 0.00938825 | 0.00085362 | 100 | 20 |
| HC | 1280 | G | T | 0.005668704 | 0.000745303 | 100 | 20 |
| HC | 205 | C | A | 0.000992579 | 0.001282893 | 100 | 0 |
| HC | 322 | C | A | 0.007438526 | 0.0008519 | 100 | 0 |
| HC | 531 | T | G | 0.007064722 | 0.000841497 | 100 | 0 |
| HC | 689 | A | C | 0.008756243 | 0.000517423 | 100 | 0 |
| HC | 817 | T | A | 0.000412094 | 0.00050926 | 100 | 0 |
| HC | 818 | C | A | 0.001909226 | 0.000531043 | 100 | 0 |
| HC | 910 | C | T | 0.000378335 | 0.00159492 | 100 | 0 |
| HC | 1161 | C | T | 0.001623252 | 0.0006381 | 100 | 0 |
| HC | 1203 | A | C | 0.003112853 | 0.00099937 | 100 | 0 |
| HC | 819 | C | D | 0.001362135 | 0.000809177 | 50 | 80 |
| HC | 820 | T | C | 0.000737488 | 0.00426519 | 50 | 80 |
| HC | 1052 | G | C | 0.002028774 | 0.000653137 | 50 | 80 |
| HC | 1394 | A | G | 0.000942301 | 0.00083782 | 50 | 20 |
| HC | 603 | T | D | 0.005349817 | 0.000600033 | 50 | 0 |
| HC | 817 | T | A | 0.005480119 | 0.000575353 | 50 | 0 |
| DHFR | 125 | A | G | 0.001105588 | 0.000543173 | 150 | 80 |
| DHFR | 127 | C | A | 0.000174852 | 0.00105013 | 150 | 80 |
| DHFR | 129 | A | C | 0.000328318 | 0.001275663 | 150 | 80 |
| DHFR | 132 | C | A | 0.000305323 | 0.00051187 | 150 | 80 |
| DHFR | 140 | C | A | 0.00959339 | 0.000649163 | 150 | 80 |
| DHFR | 142 | C | A | 0.000859426 | 0.000654067 | 150 | 80 |
| DHFR | 144 | C | A | 4.18E-05 | 0.00065926 | 150 | 80 |
| DHFR | 148 | A | C | 0.000471312 | 0.000683317 | 150 | 80 |
| DHFR | 149 | A | C | 0.000284372 | 0.00056614 | 150 | 80 |
| DHFR | 152 | A | C | 0.000487242 | 0.00069889 | 150 | 80 |
| DHFR | 161 | A | C | 2.02E-05 | 0.001260067 | 150 | 80 |
| DHFR | 162 | C | A | 0.003120878 | 0.001339697 | 150 | 80 |
| DHFR | 165 | C | A | 0.000491153 | 0.001700553 | 150 | 80 |
| DHFR | 166 | C | A | 6.58E-05 | 0.001384123 | 150 | 80 |
| DHFR | 167 | A | C | 0.001899001 | 0.00082739 | 150 | 80 |
| DHFR | 168 | A | C | 0.00924121 | 0.00074645 | 150 | 80 |
| DHFR | 169 | A | C | 0.000141375 | 0.000737007 | 150 | 80 |
| DHFR | 172 | A | C | 0.001265005 | 0.000865313 | 150 | 80 |
| DHFR | 175 | A | C | 0.001822178 | 0.001591397 | 150 | 80 |
| DHFR | 176 | C | A | 0.002024222 | 0.002009133 | 150 | 80 |
| DHFR | 177 | C | A | 0.001007817 | 0.00115327 | 150 | 80 |
| DHFR | 178 | A | C | 0.00030024 | 0.00130025 | 150 | 80 |
| DHFR | 179 | C | A | 0.008392894 | 0.001184187 | 150 | 80 |
| DHFR | 180 | A | C | 0.00251627 | 0.000872053 | 150 | 80 |
| DHFR | 181 | A | C | 0.004009001 | 0.001881253 | 150 | 80 |
| DHFR | 182 | C | A | 0.002391624 | 0.001958427 | 150 | 80 |
| DHFR | 183 | C | A | 0.000227924 | 0.00135707 | 150 | 80 |
| DHFR | 184 | T | A | 0.000277634 | 0.002036853 | 150 | 80 |
| DHFR | 185 | C | A | 0.003345879 | 0.001055757 | 150 | 80 |
| DHFR | 196 | G | A | 0.00409521 | 0.00142319 | 150 | 80 |
| DHFR | 197 | G | A | 0.006628718 | 0.000925167 | 150 | 80 |
| DHFR | 201 | A | C | 0.001806606 | 0.001267917 | 150 | 80 |
| DHFR | 205 | A | C | 1.24E-05 | 0.00075653 | 150 | 80 |
| DHFR | 220 | G | T | 0.000109323 | 0.000987977 | 150 | 80 |
| DHFR | 233 | G | T | 1.03E-05 | 0.001305083 | 150 | 80 |
| DHFR | 257 | G | T | 0.000390879 | 0.001064217 | 150 | 80 |
| DHFR | 272 | G | T | 8.32E-05 | 0.000848337 | 150 | 80 |
| DHFR | 279 | T | G | 0.00275608 | 0.000519247 | 150 | 80 |
| DHFR | 293 | G | T | 0.008483882 | 0.0010318 | 150 | 80 |
| DHFR | 295 | G | T | 0.008803628 | 0.000716463 | 150 | 80 |
| DHFR | 301 | A | G | 0.005853317 | 0.000604553 | 150 | 80 |
| DHFR | 331 | G | T | 0.002747957 | 0.00120216 | 150 | 80 |
| DHFR | 339 | T | G | 1.29E-05 | 0.000917117 | 150 | 80 |
| DHFR | 342 | G | T | 0.002117813 | 0.000669397 | 150 | 80 |
| DHFR | 343 | G | T | 0.001088305 | 0.000866687 | 150 | 80 |
| DHFR | 349 | G | T | 0.002132306 | 0.000868493 | 150 | 80 |
| DHFR | 383 | G | T | 0.000140607 | 0.00083814 | 150 | 80 |
| DHFR | 425 | A | C | 6.10E-05 | 0.001025453 | 150 | 80 |
| DHFR | 435 | C | A | 0.00581927 | 0.00127045 | 150 | 80 |
| DHFR | 446 | C | A | 0.000309457 | 0.00093123 | 150 | 80 |
| DHFR | 450 | C | A | 0.004002571 | 0.000802617 | 150 | 80 |
| DHFR | 451 | C | A | 0.000131246 | 0.000720177 | 150 | 80 |
| DHFR | 452 | A | C | 0.000253261 | 0.002039273 | 150 | 80 |
| DHFR | 454 | C | A | 0.001131568 | 0.000844463 | 150 | 80 |
| DHFR | 459 | A | C | 0.007391094 | 0.001552153 | 150 | 80 |
| DHFR | 460 | C | A | 0.006035591 | 0.00114726 | 150 | 80 |
| DHFR | 462 | C | A | 0.009188285 | 0.001015447 | 150 | 80 |
| DHFR | 470 | C | A | 0.005038331 | 0.001178677 | 150 | 80 |
| DHFR | 475 | A | C | 0.007116874 | 0.00081589 | 150 | 80 |
| DHFR | 485 | A | C | 0.002492344 | 0.00070603 | 150 | 80 |
| DHFR | 489 | T | C | 0.009126208 | 0.000560933 | 150 | 80 |
| DHFR | 498 | C | A | 0.001553755 | 0.001059307 | 150 | 80 |
| DHFR | 500 | C | A | 0.003587002 | 0.00095218 | 150 | 80 |
| DHFR | 507 | C | A | 0.006038463 | 0.00104162 | 150 | 80 |
| DHFR | 508 | C | A | 0.00915518 | 0.001672977 | 150 | 80 |
| DHFR | 509 | C | A | 0.005211747 | 0.000897467 | 150 | 80 |
| DHFR | 515 | T | G | 0.00135633 | 0.000884403 | 150 | 80 |
| DHFR | 516 | T | G | 0.004856328 | 0.000684747 | 150 | 80 |
| DHFR | 517 | G | T | 0.005713264 | 0.001607397 | 150 | 80 |
| DHFR | 519 | T | G | 0.001477345 | 0.000833333 | 150 | 80 |
| DHFR | 521 | T | G | 0.000149966 | 0.0009734 | 150 | 80 |
| DHFR | 523 | G | T | 0.001014997 | 0.001551323 | 150 | 80 |
| DHFR | 524 | G | T | 0.001571757 | 0.002215713 | 150 | 80 |
| DHFR | 525 | G | T | 0.006885614 | 0.001986503 | 150 | 80 |
| DHFR | 526 | A | T | 0.00301353 | 0.000886077 | 150 | 80 |
| DHFR | 529 | T | G | 3.39E-05 | 0.001009253 | 150 | 80 |
| DHFR | 531 | T | G | 0.003921319 | 0.00074962 | 150 | 80 |
| DHFR | 536 | T | G | 0.000257428 | 0.000806557 | 150 | 80 |
| DHFR | 537 | T | G | 0.006314558 | 0.000691653 | 150 | 80 |
| DHFR | 539 | T | G | 0.004757801 | 0.000755967 | 150 | 80 |
| DHFR | 544 | G | T | 0.000397674 | 0.001206347 | 150 | 80 |
| DHFR | 547 | T | G | 0.005253099 | 0.000835177 | 150 | 80 |
| DHFR | 557 | T | G | 0.005990433 | 0.00081562 | 150 | 80 |
| DHFR | 563 | C | A | 2.07E-05 | 0.0198197 | 150 | 80 |
| DHFR | 565 | G | T | 0.001408028 | 0.000665977 | 150 | 80 |
| DHFR | 567 | G | T | 0.006265433 | 0.0006164 | 150 | 80 |
| DHFR | 569 | T | G | 0.00104303 | 0.00086948 | 150 | 80 |
| DHFR | 573 | G | T | 0.005606683 | 0.000715453 | 150 | 80 |
| DHFR | 81 | C | T | 0.002862837 | 0.00312021 | 150 | 20 |
| DHFR | 132 | C | A | 0.000252472 | 0.000507 | 150 | 20 |
| DHFR | 144 | C | A | 0.006597308 | 0.000641683 | 150 | 20 |
| DHFR | 148 | A | C | 0.005207239 | 0.00055549 | 150 | 20 |
| DHFR | 149 | A | C | 0.003687588 | 0.0006277 | 150 | 20 |
| DHFR | 152 | A | C | 0.001206985 | 0.00053279 | 150 | 20 |
| DHFR | 161 | A | C | 0.002697047 | 0.001210153 | 150 | 20 |
| DHFR | 162 | C | A | 0.001183832 | 0.001323877 | 150 | 20 |
| DHFR | 165 | C | A | 1.21E-05 | 0.00141554 | 150 | 20 |
| DHFR | 166 | C | A | 5.82E-05 | 0.00130059 | 150 | 20 |
| DHFR | 167 | A | C | 5.57E-06 | 0.00073377 | 150 | 20 |
| DHFR | 168 | A | C | 0.001402467 | 0.000820413 | 150 | 20 |
| DHFR | 169 | A | C | 0.002394078 | 0.000741683 | 150 | 20 |
| DHFR | 171 | A | C | 0.000265646 | 0.00090378 | 150 | 20 |
| DHFR | 172 | A | C | 0.001053965 | 0.000764283 | 150 | 20 |
| DHFR | 175 | A | C | 0.000218409 | 0.00147457 | 150 | 20 |
| DHFR | 176 | C | A | 0.005044548 | 0.001772557 | 150 | 20 |
| DHFR | 177 | C | A | 0.001392152 | 0.001300247 | 150 | 20 |
| DHFR | 178 | A | C | 0.002846288 | 0.00116374 | 150 | 20 |
| DHFR | 179 | C | A | 0.001027262 | 0.001600167 | 150 | 20 |
| DHFR | 181 | A | C | 0.004157752 | 0.001547383 | 150 | 20 |
| DHFR | 182 | C | A | 0.004887183 | 0.00140531 | 150 | 20 |
| DHFR | 183 | C | A | 8.26E-06 | 0.00092795 | 150 | 20 |
| DHFR | 184 | T | A | 0.002689282 | 0.00213615 | 150 | 20 |
| DHFR | 188 | C | A | 0.0066642 | 0.000877663 | 150 | 20 |
| DHFR | 189 | A | G | 0.001828008 | 0.000501363 | 150 | 20 |
| DHFR | 190 | G | A | 0.003094459 | 0.00064793 | 150 | 20 |
| DHFR | 192 | G | A | 0.002539877 | 0.000617093 | 150 | 20 |
| DHFR | 197 | G | A | 0.003560615 | 0.002969367 | 150 | 20 |
| DHFR | 200 | A | C | 0.004042222 | 0.000710427 | 150 | 20 |
| DHFR | 210 | G | A | 0.005151983 | 0.00090431 | 150 | 20 |
| DHFR | 225 | G | T | 0.002301002 | 0.000773673 | 150 | 20 |
| DHFR | 257 | G | T | 0.003620801 | 0.00103197 | 150 | 20 |
| DHFR | 267 | G | T | 0.001224669 | 0.000670583 | 150 | 20 |
| DHFR | 268 | G | T | 0.002235473 | 0.0008468 | 150 | 20 |
| DHFR | 272 | G | T | 0.006754665 | 0.00074739 | 150 | 20 |
| DHFR | 283 | G | T | 0.008861742 | 0.000767727 | 150 | 20 |
| DHFR | 284 | T | G | 0.003841308 | 0.00097232 | 150 | 20 |
| DHFR | 295 | G | T | 0.001286788 | 0.000689013 | 150 | 20 |
| DHFR | 316 | G | T | 0.00784309 | 0.000546223 | 150 | 20 |
| DHFR | 342 | G | T | 0.000274782 | 0.000836843 | 150 | 20 |
| DHFR | 346 | G | T | 0.002813328 | 0.00082586 | 150 | 20 |
| DHFR | 373 | G | T | 0.004794254 | 0.000546273 | 150 | 20 |
| DHFR | 402 | G | A | 0.003855389 | 0.000508037 | 150 | 20 |
| DHFR | 408 | C | A | 0.001352555 | 0.001138963 | 150 | 20 |
| DHFR | 414 | C | A | 0.009537313 | 0.000886807 | 150 | 20 |
| DHFR | 435 | C | A | 0.000175005 | 0.00100675 | 150 | 20 |
| DHFR | 442 | C | A | 0.005739773 | 0.001129187 | 150 | 20 |
| DHFR | 446 | C | A | 0.000819658 | 0.00098206 | 150 | 20 |
| DHFR | 452 | A | C | 0.004553948 | 0.001961743 | 150 | 20 |
| DHFR | 453 | C | A | 0.006363744 | 0.00129317 | 150 | 20 |
| DHFR | 470 | C | A | 0.00028918 | 0.000951377 | 150 | 20 |
| DHFR | 477 | C | A | 0.002245602 | 0.000669647 | 150 | 20 |
| DHFR | 486 | A | C | 0.002900884 | 0.00077206 | 150 | 20 |
| DHFR | 499 | A | C | 0.007048013 | 0.0011394 | 150 | 20 |
| DHFR | 500 | C | A | 0.003854553 | 0.000781577 | 150 | 20 |
| DHFR | 508 | C | A | 0.001871153 | 0.00129958 | 150 | 20 |
| DHFR | 515 | T | G | 0.0027591 | 0.0008521 | 150 | 20 |
| DHFR | 516 | T | G | 0.003371642 | 0.000753963 | 150 | 20 |
| DHFR | 517 | G | T | 0.004911179 | 0.001701157 | 150 | 20 |
| DHFR | 519 | T | G | 0.000371473 | 0.00121101 | 150 | 20 |
| DHFR | 520 | T | G | 0.000171911 | 0.0010793 | 150 | 20 |
| DHFR | 521 | T | G | 0.00045813 | 0.000788663 | 150 | 20 |
| DHFR | 523 | G | T | 6.98E-05 | 0.001657667 | 150 | 20 |
| DHFR | 524 | G | T | 0.001902324 | 0.00187113 | 150 | 20 |
| DHFR | 525 | G | T | 0.000614951 | 0.001581247 | 150 | 20 |
| DHFR | 526 | A | T | 0.005089222 | 0.000842023 | 150 | 20 |
| DHFR | 529 | T | G | 0.00949336 | 0.000958597 | 150 | 20 |
| DHFR | 539 | T | G | 0.005291755 | 0.000686183 | 150 | 20 |
| DHFR | 544 | G | T | 0.001875529 | 0.001218703 | 150 | 20 |
| DHFR | 547 | T | G | 0.008338357 | 0.000594207 | 150 | 20 |
| DHFR | 553 | G | T | 0.006039267 | 0.000701487 | 150 | 20 |
| DHFR | 554 | G | T | 0.007541351 | 0.00073086 | 150 | 20 |
| DHFR | 565 | G | T | 0.002136335 | 0.000507193 | 150 | 20 |
| DHFR | 569 | T | G | 0.009681063 | 0.000683477 | 150 | 20 |
| DHFR | 573 | G | T | 0.008318001 | 0.00064215 | 150 | 20 |
| DHFR | 125 | A | G | 0.000587887 | 0.00056381 | 150 | 0 |
| DHFR | 132 | C | A | 0.004096312 | 0.000557973 | 150 | 0 |
| DHFR | 161 | A | C | 0.006104169 | 0.001619463 | 150 | 0 |
| DHFR | 162 | C | A | 0.000762413 | 0.001319567 | 150 | 0 |
| DHFR | 168 | A | C | 0.003791769 | 0.000908567 | 150 | 0 |
| DHFR | 169 | A | C | 0.000823882 | 0.00078374 | 150 | 0 |
| DHFR | 171 | A | C | 0.001004842 | 0.000813807 | 150 | 0 |
| DHFR | 172 | A | C | 0.002079546 | 0.00087243 | 150 | 0 |
| DHFR | 175 | A | C | 0.001384545 | 0.001610533 | 150 | 0 |
| DHFR | 177 | C | A | 0.003011643 | 0.001016967 | 150 | 0 |
| DHFR | 178 | A | C | 0.007425152 | 0.001436743 | 150 | 0 |
| DHFR | 179 | C | A | 0.005847904 | 0.001455397 | 150 | 0 |
| DHFR | 181 | A | C | 0.004806059 | 0.001769183 | 150 | 0 |
| DHFR | 182 | C | A | 0.000233874 | 0.001591623 | 150 | 0 |
| DHFR | 183 | C | A | 0.000695545 | 0.00114447 | 150 | 0 |
| DHFR | 184 | T | A | 0.005789596 | 0.001602623 | 150 | 0 |
| DHFR | 185 | C | A | 0.006311984 | 0.000854757 | 150 | 0 |
| DHFR | 187 | T | A | 0.006689821 | 0.00108836 | 150 | 0 |
| DHFR | 202 | C | A | 0.002088614 | 0.001146763 | 150 | 0 |
| DHFR | 219 | G | T | 0.008713775 | 0.001069833 | 150 | 0 |
| DHFR | 220 | G | T | 0.000212536 | 0.00098718 | 150 | 0 |
| DHFR | 224 | G | T | 0.000143192 | 0.000895837 | 150 | 0 |
| DHFR | 225 | G | T | 0.000947733 | 0.001264153 | 150 | 0 |
| DHFR | 233 | G | T | 0.002765337 | 0.00101441 | 150 | 0 |
| DHFR | 234 | G | T | 0.007803152 | 0.001200717 | 150 | 0 |
| DHFR | 240 | C | T | 0.000125705 | 0.00051225 | 150 | 0 |
| DHFR | 247 | G | T | 0.007014445 | 0.001234367 | 150 | 0 |
| DHFR | 249 | G | T | 0.006335492 | 0.00055296 | 150 | 0 |
| DHFR | 268 | G | T | 0.004749629 | 0.001233877 | 150 | 0 |
| DHFR | 319 | G | T | 0.000491324 | 0.000793183 | 150 | 0 |
| DHFR | 331 | G | T | 0.007725949 | 0.000953763 | 150 | 0 |
| DHFR | 332 | C | T | 0.002069941 | 0.00070756 | 150 | 0 |
| DHFR | 338 | G | T | 0.001321286 | 0.00080385 | 150 | 0 |
| DHFR | 339 | T | G | 0.001383476 | 0.001127017 | 150 | 0 |
| DHFR | 342 | G | T | 0.001832226 | 0.000956783 | 150 | 0 |
| DHFR | 349 | G | T | 0.003959038 | 0.001012037 | 150 | 0 |
| DHFR | 392 | A | C | 6.22E-05 | 0.000855237 | 150 | 0 |
| DHFR | 393 | C | A | 0.00542977 | 0.00106885 | 150 | 0 |
| DHFR | 408 | C | A | 0.001590599 | 0.000813743 | 150 | 0 |
| DHFR | 435 | C | A | 0.003815955 | 0.00082469 | 150 | 0 |
| DHFR | 442 | C | A | 0.005486693 | 0.000857927 | 150 | 0 |
| DHFR | 446 | C | A | 0.003105662 | 0.00083945 | 150 | 0 |
| DHFR | 454 | C | A | 0.009855368 | 0.000948353 | 150 | 0 |
| DHFR | 460 | C | A | 0.004332952 | 0.00055436 | 150 | 0 |
| DHFR | 470 | C | A | 0.009389658 | 0.000939087 | 150 | 0 |
| DHFR | 474 | G | A | 0.001705689 | 0.000532567 | 150 | 0 |
| DHFR | 475 | A | C | 0.004447853 | 0.000873313 | 150 | 0 |
| DHFR | 500 | C | A | 0.000654399 | 0.000916977 | 150 | 0 |
| DHFR | 507 | C | A | 0.009357755 | 0.000949617 | 150 | 0 |
| DHFR | 509 | C | A | 0.002221018 | 0.001160283 | 150 | 0 |
| DHFR | 515 | T | G | 0.000839542 | 0.000828497 | 150 | 0 |
| DHFR | 517 | G | T | 0.001634784 | 0.00161272 | 150 | 0 |
| DHFR | 519 | T | G | 0.008417222 | 0.001109443 | 150 | 0 |
| DHFR | 520 | T | G | 0.002483225 | 0.001042147 | 150 | 0 |
| DHFR | 521 | T | G | 0.000546565 | 0.000761753 | 150 | 0 |
| DHFR | 524 | G | T | 0.00402268 | 0.00198936 | 150 | 0 |
| DHFR | 525 | G | T | 0.003473739 | 0.001535777 | 150 | 0 |
| DHFR | 531 | T | G | 0.006608723 | 0.000840367 | 150 | 0 |
| DHFR | 536 | T | G | 0.009981344 | 0.000767023 | 150 | 0 |
| DHFR | 537 | T | G | 0.007100123 | 0.00075103 | 150 | 0 |
| DHFR | 539 | T | G | 0.004391698 | 0.00087942 | 150 | 0 |
| DHFR | 553 | G | T | 0.006043724 | 0.00108775 | 150 | 0 |
| DHFR | 129 | A | C | 0.002543844 | 0.00060994 | 100 | 80 |
| DHFR | 563 | C | A | 7.17E-05 | 0.000549193 | 100 | 80 |
| DHFR | 184 | T | A | 0.000145617 | 0.00120717 | 100 | 20 |
| DHFR | 187 | T | A | 0.000487878 | 0.001563163 | 100 | 20 |
| DHFR | 518 | A | T | 0.001255254 | 0.00114911 | 100 | 20 |
| DHFR | 526 | A | T | 0.000906604 | 0.000660817 | 100 | 20 |
| DHFR | 129 | A | C | 0.000556003 | 0.000760803 | 100 | 0 |
| DHFR | 184 | T | A | 0.00175381 | 0.00092436 | 100 | 0 |
| DHFR | 196 | G | A | 0.004370895 | 0.000529773 | 100 | 0 |
| DHFR | 518 | A | T | 0.00107742 | 0.000935917 | 100 | 0 |
| DHFR | 451 | C | A | 0.009464289 | 0.001352303 | 50 | 20 |
| GAPDH | 65 | T | C | 0.0080403 | 0.000731987 | 150 | 80 |
| GAPDH | 109 | A | G | 0.002831669 | 0.000570193 | 150 | 80 |
| GAPDH | 153 | C | A | 0.000483798 | 0.000781623 | 150 | 80 |
| GAPDH | 158 | A | C | 0.000943921 | 0.001776587 | 150 | 80 |
| GAPDH | 159 | C | A | 0.00253314 | 0.00121948 | 150 | 80 |
| GAPDH | 160 | C | A | 0.003067869 | 0.00095238 | 150 | 80 |
| GAPDH | 161 | C | A | 0.001503732 | 0.00114956 | 150 | 80 |
| GAPDH | 162 | C | A | 0.005661108 | 0.000844333 | 150 | 80 |
| GAPDH | 165 | C | A | 0.001057221 | 0.001216043 | 150 | 80 |
| GAPDH | 166 | A | C | 0.007914329 | 0.000689057 | 150 | 80 |
| GAPDH | 170 | A | C | 7.26E-05 | 0.001497543 | 150 | 80 |
| GAPDH | 171 | C | A | 0.000499626 | 0.00169072 | 150 | 80 |
| GAPDH | 172 | C | A | 0.000211918 | 0.001340213 | 150 | 80 |
| GAPDH | 174 | C | A | 0.002536174 | 0.000993617 | 150 | 80 |
| GAPDH | 176 | A | C | 0.000350494 | 0.001632313 | 150 | 80 |
| GAPDH | 177 | C | A | 3.54E-05 | 0.001238693 | 150 | 80 |
| GAPDH | 180 | C | A | 0.00541446 | 0.001965883 | 150 | 80 |
| GAPDH | 181 | A | C | 0.004398233 | 0.000826063 | 150 | 80 |
| GAPDH | 186 | C | A | 0.000623172 | 0.001204137 | 150 | 80 |
| GAPDH | 188 | A | C | 0.0038952 | 0.00157123 | 150 | 80 |
| GAPDH | 189 | C | A | 0.008613333 | 0.001479847 | 150 | 80 |
| GAPDH | 190 | A | G | 0.005825765 | 0.0005752 | 150 | 80 |
| GAPDH | 195 | C | A | 0.009683321 | 0.001951143 | 150 | 80 |
| GAPDH | 198 | G | A | 0.001492864 | 0.001609757 | 150 | 80 |
| GAPDH | 202 | G | T | 0.000280324 | 0.000826787 | 150 | 80 |
| GAPDH | 226 | G | T | 0.009447969 | 0.000603433 | 150 | 80 |
| GAPDH | 247 | G | T | 0.008232275 | 0.000793183 | 150 | 80 |
| GAPDH | 248 | G | T | 0.001171297 | 0.001187437 | 150 | 80 |
| GAPDH | 265 | G | T | 0.003238176 | 0.000821303 | 150 | 80 |
| GAPDH | 266 | G | T | 0.00240939 | 0.000763837 | 150 | 80 |
| GAPDH | 271 | G | T | 0.007323202 | 0.001037347 | 150 | 80 |
| GAPDH | 273 | C | T | 0.001244232 | 0.000623017 | 150 | 80 |
| GAPDH | 293 | G | T | 0.003872276 | 0.000614857 | 150 | 80 |
| GAPDH | 295 | G | T | 3.03E-05 | 0.000569727 | 150 | 80 |
| GAPDH | 297 | T | G | 0.002002973 | 0.000576847 | 150 | 80 |
| GAPDH | 299 | C | T | 0.006022491 | 0.000597947 | 150 | 80 |
| GAPDH | 303 | C | A | 0.003218086 | 0.000650673 | 150 | 80 |
| GAPDH | 306 | C | A | 0.00880167 | 0.000702607 | 150 | 80 |
| GAPDH | 329 | C | A | 4.36E-06 | 0.000894853 | 150 | 80 |
| GAPDH | 330 | C | A | 0.000956672 | 0.00092733 | 150 | 80 |
| GAPDH | 349 | A | C | 0.000648064 | 0.001005327 | 150 | 80 |
| GAPDH | 357 | C | A | 0.000326728 | 0.000684473 | 150 | 80 |
| GAPDH | 363 | C | A | 0.003761749 | 0.000597503 | 150 | 80 |
| GAPDH | 383 | C | A | 0.00266067 | 0.000853573 | 150 | 80 |
| GAPDH | 386 | A | C | 0.007192193 | 0.0015327 | 150 | 80 |
| GAPDH | 408 | G | T | 0.004261073 | 0.000835687 | 150 | 80 |
| GAPDH | 433 | G | T | 0.004503968 | 0.000962957 | 150 | 80 |
| GAPDH | 436 | G | T | 0.00047335 | 0.000679657 | 150 | 80 |
| GAPDH | 443 | T | G | 0.003732329 | 0.00064035 | 150 | 80 |
| GAPDH | 444 | G | T | 0.008419299 | 0.00105271 | 150 | 80 |
| GAPDH | 445 | T | G | 0.003958114 | 0.000584173 | 150 | 80 |
| GAPDH | 450 | G | T | 0.002350619 | 0.000816287 | 150 | 80 |
| GAPDH | 453 | G | T | 0.003795224 | 0.000929103 | 150 | 80 |
| GAPDH | 454 | G | T | 0.000589895 | 0.000980497 | 150 | 80 |
| GAPDH | 459 | G | T | 0.000317281 | 0.000508487 | 150 | 80 |
| GAPDH | 466 | G | T | 0.00060428 | 0.00064404 | 150 | 80 |
| GAPDH | 471 | G | T | 0.007212309 | 0.000553433 | 150 | 80 |
| GAPDH | 472 | T | G | 1.40E-05 | 0.001114777 | 150 | 80 |
| GAPDH | 489 | G | T | 0.007789401 | 0.00078949 | 150 | 80 |
| GAPDH | 498 | C | T | 0.009506416 | 0.000546187 | 150 | 80 |
| GAPDH | 503 | C | T | 0.001078486 | 0.0006192 | 150 | 80 |
| GAPDH | 505 | T | G | 0.001405551 | 0.000611763 | 150 | 80 |
| GAPDH | 512 | C | T | 0.009562138 | 0.000525817 | 150 | 80 |
| GAPDH | 530 | C | A | 0.002261589 | 0.00067207 | 150 | 80 |
| GAPDH | 531 | C | A | 0.000238455 | 0.00064046 | 150 | 80 |
| GAPDH | 532 | C | A | 0.001379095 | 0.000777273 | 150 | 80 |
| GAPDH | 536 | C | A | 0.007312587 | 0.00082428 | 150 | 80 |
| GAPDH | 543 | C | A | 0.006069523 | 0.000532177 | 150 | 80 |
| GAPDH | 550 | G | T | 0.007500059 | 0.000827623 | 150 | 80 |
| GAPDH | 559 | G | T | 0.000575483 | 0.000604453 | 150 | 80 |
| GAPDH | 560 | G | T | 0.002491806 | 0.000959267 | 150 | 80 |
| GAPDH | 579 | G | T | 0.00699213 | 0.000723427 | 150 | 80 |
| GAPDH | 627 | C | A | 0.004081414 | 0.000698647 | 150 | 80 |
| GAPDH | 629 | C | A | 0.000256754 | 0.00102817 | 150 | 80 |
| GAPDH | 652 | C | A | 0.001012364 | 0.000743133 | 150 | 80 |
| GAPDH | 680 | C | T | 0.00579608 | 0.00064269 | 150 | 80 |
| GAPDH | 688 | G | T | 0.008521634 | 0.00071779 | 150 | 80 |
| GAPDH | 690 | C | T | 0.007808646 | 0.000572393 | 150 | 80 |
| GAPDH | 703 | G | T | 0.003402949 | 0.000871487 | 150 | 80 |
| GAPDH | 706 | G | T | 0.007784117 | 0.000851333 | 150 | 80 |
| GAPDH | 713 | T | G | 0.002244168 | 0.000917007 | 150 | 80 |
| GAPDH | 726 | G | T | 0.006756819 | 0.000922787 | 150 | 80 |
| GAPDH | 730 | G | T | 0.005722987 | 0.000787013 | 150 | 80 |
| GAPDH | 735 | G | T | 0.006982171 | 0.000542687 | 150 | 80 |
| GAPDH | 742 | G | T | 0.003324545 | 0.000590687 | 150 | 80 |
| GAPDH | 747 | G | T | 0.003451125 | 0.000669623 | 150 | 80 |
| GAPDH | 761 | C | A | 0.003491017 | 0.000611513 | 150 | 80 |
| GAPDH | 765 | C | A | 0.008234125 | 0.000689527 | 150 | 80 |
| GAPDH | 766 | C | A | 0.000375363 | 0.000571373 | 150 | 80 |
| GAPDH | 768 | C | A | 0.001783304 | 0.000623333 | 150 | 80 |
| GAPDH | 770 | A | C | 0.004764098 | 0.000878677 | 150 | 80 |
| GAPDH | 787 | C | A | 0.000829516 | 0.000725247 | 150 | 80 |
| GAPDH | 799 | C | A | 0.007263174 | 0.00074232 | 150 | 80 |
| GAPDH | 808 | C | A | 0.00179336 | 0.000641507 | 150 | 80 |
| GAPDH | 813 | C | A | 0.003328545 | 0.00089104 | 150 | 80 |
| GAPDH | 818 | A | C | 0.009747865 | 0.0007142 | 150 | 80 |
| GAPDH | 837 | G | A | 8.64E-05 | 0.000558537 | 150 | 80 |
| GAPDH | 847 | G | A | 0.005018376 | 0.000553033 | 150 | 80 |
| GAPDH | 859 | C | A | 0.003923708 | 0.000827077 | 150 | 80 |
| GAPDH | 860 | C | A | 0.002656024 | 0.00082596 | 150 | 80 |
| GAPDH | 861 | A | C | 0.00903973 | 0.00150649 | 150 | 80 |
| GAPDH | 869 | G | A | 0.000101086 | 0.00066197 | 150 | 80 |
| GAPDH | 870 | C | A | 0.001790028 | 0.000670357 | 150 | 80 |
| GAPDH | 873 | C | A | 0.007697028 | 0.000719287 | 150 | 80 |
| GAPDH | 874 | C | A | 0.007956426 | 0.000667447 | 150 | 80 |
| GAPDH | 878 | G | A | 0.002426904 | 0.000516057 | 150 | 80 |
| GAPDH | 879 | C | A | 0.003612455 | 0.000699537 | 150 | 80 |
| GAPDH | 885 | C | A | 0.002659519 | 0.001079553 | 150 | 80 |
| GAPDH | 892 | C | A | 0.001504771 | 0.00085651 | 150 | 80 |
| GAPDH | 905 | G | A | 0.001765478 | 0.000543823 | 150 | 80 |
| GAPDH | 908 | A | C | 0.00321848 | 0.001229813 | 150 | 80 |
| GAPDH | 909 | C | A | 0.003261354 | 0.000833813 | 150 | 80 |
| GAPDH | 921 | C | A | 0.000434421 | 0.000656677 | 150 | 80 |
| GAPDH | 926 | A | C | 0.000110082 | 0.0019237 | 150 | 80 |
| GAPDH | 927 | C | A | 0.000674036 | 0.000703613 | 150 | 80 |
| GAPDH | 929 | C | A | 0.000183568 | 0.000704993 | 150 | 80 |
| GAPDH | 934 | A | G | 0.002311972 | 0.001658077 | 150 | 80 |
| GAPDH | 949 | G | A | 0.007506452 | 0.00062532 | 150 | 80 |
| GAPDH | 950 | C | A | 0.008443423 | 0.00069444 | 150 | 80 |
| GAPDH | 953 | G | A | 0.007184749 | 0.000894323 | 150 | 80 |
| GAPDH | 961 | C | A | 0.006082473 | 0.00079086 | 150 | 80 |
| GAPDH | 969 | C | A | 0.005151683 | 0.000937097 | 150 | 80 |
| GAPDH | 971 | A | C | 0.001798884 | 0.001210107 | 150 | 80 |
| GAPDH | 972 | C | A | 0.002661601 | 0.000736947 | 150 | 80 |
| GAPDH | 982 | C | A | 0.000953414 | 0.000566427 | 150 | 80 |
| GAPDH | 989 | C | A | 5.26E-05 | 0.000723503 | 150 | 80 |
| GAPDH | 1005 | A | C | 0.007175718 | 0.000528743 | 150 | 80 |
| GAPDH | 1014 | C | A | 0.006259787 | 0.000950137 | 150 | 80 |
| GAPDH | 1017 | C | A | 0.000467029 | 0.000680827 | 150 | 80 |
| GAPDH | 1018 | A | C | 0.000462933 | 0.000773267 | 150 | 80 |
| GAPDH | 1019 | A | C | 0.006551485 | 0.001020347 | 150 | 80 |
| GAPDH | 1031 | A | C | 0.001715906 | 0.00089991 | 150 | 80 |
| GAPDH | 1049 | C | A | 0.005650675 | 0.00084157 | 150 | 80 |
| GAPDH | 65 | T | C | 0.000562716 | 0.00078953 | 150 | 20 |
| GAPDH | 71 | T | C | 8.95E-05 | 0.0007468 | 150 | 20 |
| GAPDH | 153 | C | A | 0.000376861 | 0.00086587 | 150 | 20 |
| GAPDH | 158 | A | C | 0.009363341 | 0.001546303 | 150 | 20 |
| GAPDH | 159 | C | A | 0.001325874 | 0.001057063 | 150 | 20 |
| GAPDH | 160 | C | A | 0.009438426 | 0.00066518 | 150 | 20 |
| GAPDH | 161 | C | A | 0.002165378 | 0.000993137 | 150 | 20 |
| GAPDH | 162 | C | A | 0.00897932 | 0.000748907 | 150 | 20 |
| GAPDH | 165 | C | A | 4.60E-06 | 0.001079177 | 150 | 20 |
| GAPDH | 166 | A | C | 3.14E-05 | 0.000611303 | 150 | 20 |
| GAPDH | 170 | A | C | 0.004539559 | 0.001442727 | 150 | 20 |
| GAPDH | 171 | C | A | 0.001063834 | 0.00194285 | 150 | 20 |
| GAPDH | 172 | C | A | 6.58E-05 | 0.00130919 | 150 | 20 |
| GAPDH | 174 | C | A | 0.000140348 | 0.001169797 | 150 | 20 |
| GAPDH | 176 | A | C | 0.00536326 | 0.001661127 | 150 | 20 |
| GAPDH | 180 | C | A | 0.001606208 | 0.001527313 | 150 | 20 |
| GAPDH | 181 | A | C | 0.003106133 | 0.00117925 | 150 | 20 |
| GAPDH | 186 | C | A | 0.005074742 | 0.00156727 | 150 | 20 |
| GAPDH | 189 | C | A | 0.009010711 | 0.00206288 | 150 | 20 |
| GAPDH | 190 | A | G | 0.006861088 | 0.00051506 | 150 | 20 |
| GAPDH | 195 | C | A | 0.001089236 | 0.002067227 | 150 | 20 |
| GAPDH | 197 | A | T | 0.00523195 | 0.00084509 | 150 | 20 |
| GAPDH | 199 | T | A | 0.000693694 | 0.000602867 | 150 | 20 |
| GAPDH | 202 | G | T | 0.002465886 | 0.000806657 | 150 | 20 |
| GAPDH | 214 | G | T | 0.001223288 | 0.001047893 | 150 | 20 |
| GAPDH | 219 | G | T | 0.005517428 | 0.000837683 | 150 | 20 |
| GAPDH | 237 | G | T | 0.004919083 | 0.000906597 | 150 | 20 |
| GAPDH | 241 | G | T | 0.002606524 | 0.000721373 | 150 | 20 |
| GAPDH | 248 | G | T | 0.002810652 | 0.00080658 | 150 | 20 |
| GAPDH | 256 | G | T | 0.007045278 | 0.000952477 | 150 | 20 |
| GAPDH | 267 | G | T | 0.001717258 | 0.000723393 | 150 | 20 |
| GAPDH | 271 | G | T | 0.006008935 | 0.000773687 | 150 | 20 |
| GAPDH | 289 | G | T | 0.005199679 | 0.00083957 | 150 | 20 |
| GAPDH | 295 | G | T | 0.004422757 | 0.000716397 | 150 | 20 |
| GAPDH | 297 | T | G | 0.003861825 | 0.000638427 | 150 | 20 |
| GAPDH | 302 | C | A | 0.006772926 | 0.000849033 | 150 | 20 |
| GAPDH | 306 | C | A | 0.003575031 | 0.000873647 | 150 | 20 |
| GAPDH | 329 | C | A | 5.95E-06 | 0.000863507 | 150 | 20 |
| GAPDH | 349 | A | C | 0.008792216 | 0.00098862 | 150 | 20 |
| GAPDH | 382 | G | A | 0.00896154 | 0.000567267 | 150 | 20 |
| GAPDH | 386 | A | C | 0.000263676 | 0.001200933 | 150 | 20 |
| GAPDH | 401 | C | A | 0.007159921 | 0.001318407 | 150 | 20 |
| GAPDH | 410 | T | G | 3.46E-05 | 0.000864333 | 150 | 20 |
| GAPDH | 431 | C | T | 0.009041433 | 0.000511237 | 150 | 20 |
| GAPDH | 448 | G | T | 0.003929377 | 0.000823507 | 150 | 20 |
| GAPDH | 453 | G | T | 0.000505634 | 0.000887453 | 150 | 20 |
| GAPDH | 454 | G | T | 0.001199383 | 0.00093718 | 150 | 20 |
| GAPDH | 471 | G | T | 0.001514242 | 0.000747857 | 150 | 20 |
| GAPDH | 472 | T | G | 0.001909981 | 0.001175963 | 150 | 20 |
| GAPDH | 505 | T | G | 0.004359733 | 0.00057512 | 150 | 20 |
| GAPDH | 509 | G | A | 0.005170398 | 0.00061241 | 150 | 20 |
| GAPDH | 510 | C | T | 0.004430991 | 0.000651277 | 150 | 20 |
| GAPDH | 530 | C | A | 0.007456227 | 0.00080448 | 150 | 20 |
| GAPDH | 535 | G | A | 0.002912186 | 0.00051352 | 150 | 20 |
| GAPDH | 546 | C | T | 0.000738201 | 0.000522093 | 150 | 20 |
| GAPDH | 559 | G | T | 2.03E-05 | 0.000998577 | 150 | 20 |
| GAPDH | 560 | G | T | 0.001740625 | 0.00083288 | 150 | 20 |
| GAPDH | 567 | G | T | 0.0028506 | 0.00069228 | 150 | 20 |
| GAPDH | 579 | G | T | 0.0039575 | 0.000864893 | 150 | 20 |
| GAPDH | 629 | C | A | 0.008560909 | 0.00086018 | 150 | 20 |
| GAPDH | 630 | C | A | 0.001716062 | 0.00061132 | 150 | 20 |
| GAPDH | 652 | C | A | 0.004816827 | 0.000658843 | 150 | 20 |
| GAPDH | 666 | G | T | 0.004503047 | 0.000647557 | 150 | 20 |
| GAPDH | 706 | G | T | 0.003710101 | 0.00070283 | 150 | 20 |
| GAPDH | 713 | T | G | 0.003538937 | 0.000799197 | 150 | 20 |
| GAPDH | 726 | G | T | 0.006492455 | 0.000582263 | 150 | 20 |
| GAPDH | 738 | G | T | 0.000281791 | 0.000526633 | 150 | 20 |
| GAPDH | 740 | C | T | 0.002776296 | 0.00052142 | 150 | 20 |
| GAPDH | 768 | C | A | 0.003917681 | 0.000652457 | 150 | 20 |
| GAPDH | 770 | A | C | 0.000633157 | 0.00092858 | 150 | 20 |
| GAPDH | 807 | A | C | 0.00806693 | 0.00125203 | 150 | 20 |
| GAPDH | 808 | C | A | 0.001398657 | 0.000910043 | 150 | 20 |
| GAPDH | 809 | C | A | 0.00248722 | 0.000883663 | 150 | 20 |
| GAPDH | 813 | C | A | 0.007671701 | 0.000800137 | 150 | 20 |
| GAPDH | 824 | A | C | 0.005283525 | 0.001021927 | 150 | 20 |
| GAPDH | 825 | C | A | 0.002849317 | 0.0009823 | 150 | 20 |
| GAPDH | 847 | G | A | 0.008467767 | 0.000593267 | 150 | 20 |
| GAPDH | 849 | A | C | 0.000872864 | 0.000772057 | 150 | 20 |
| GAPDH | 859 | C | A | 0.005011356 | 0.00067258 | 150 | 20 |
| GAPDH | 861 | A | C | 0.001042641 | 0.001161713 | 150 | 20 |
| GAPDH | 870 | C | A | 0.007893305 | 0.000727227 | 150 | 20 |
| GAPDH | 873 | C | A | 0.00687557 | 0.000748953 | 150 | 20 |
| GAPDH | 874 | C | A | 0.0059631 | 0.0008573 | 150 | 20 |
| GAPDH | 879 | C | A | 0.003200565 | 0.00066483 | 150 | 20 |
| GAPDH | 881 | A | C | 0.004571966 | 0.00063771 | 150 | 20 |
| GAPDH | 883 | A | G | 0.001129361 | 0.000549777 | 150 | 20 |
| GAPDH | 885 | C | A | 0.000213793 | 0.000641497 | 150 | 20 |
| GAPDH | 892 | C | A | 0.00881152 | 0.000744857 | 150 | 20 |
| GAPDH | 902 | C | A | 0.009847915 | 0.00098912 | 150 | 20 |
| GAPDH | 907 | G | A | 0.004112103 | 0.000661917 | 150 | 20 |
| GAPDH | 909 | C | A | 0.007496121 | 0.000638397 | 150 | 20 |
| GAPDH | 913 | A | G | 0.007901161 | 0.000531773 | 150 | 20 |
| GAPDH | 920 | A | C | 0.002603554 | 0.00111285 | 150 | 20 |
| GAPDH | 923 | C | A | 0.001293151 | 0.000704817 | 150 | 20 |
| GAPDH | 926 | A | C | 0.00059941 | 0.001580613 | 150 | 20 |
| GAPDH | 927 | C | A | 0.003372049 | 0.000562137 | 150 | 20 |
| GAPDH | 934 | A | G | 0.007359668 | 0.001115037 | 150 | 20 |
| GAPDH | 953 | G | A | 0.004412209 | 0.000849983 | 150 | 20 |
| GAPDH | 959 | C | A | 0.002228221 | 0.000549713 | 150 | 20 |
| GAPDH | 971 | A | C | 0.006871231 | 0.000938677 | 150 | 20 |
| GAPDH | 982 | C | A | 0.000322616 | 0.000582263 | 150 | 20 |
| GAPDH | 999 | C | A | 0.00261474 | 0.00132178 | 150 | 20 |
| GAPDH | 1011 | C | A | 0.00535828 | 0.0007966 | 150 | 20 |
| GAPDH | 1017 | C | A | 0.000492563 | 0.000558057 | 150 | 20 |
| GAPDH | 1035 | C | A | 0.009066084 | 0.00085686 | 150 | 20 |
| GAPDH | 1040 | C | A | 0.002396015 | 0.000997913 | 150 | 20 |
| GAPDH | 65 | T | C | 0.00087469 | 0.000671873 | 150 | 0 |
| GAPDH | 71 | T | C | 0.002806308 | 0.000822997 | 150 | 0 |
| GAPDH | 158 | A | C | 8.65E-07 | 0.001706777 | 150 | 0 |
| GAPDH | 170 | A | C | 9.59E-05 | 0.001368357 | 150 | 0 |
| GAPDH | 174 | C | A | 0.000229496 | 0.001003013 | 150 | 0 |
| GAPDH | 176 | A | C | 0.003039037 | 0.00127565 | 150 | 0 |
| GAPDH | 177 | C | A | 7.30E-05 | 0.00100877 | 150 | 0 |
| GAPDH | 188 | A | C | 0.00084753 | 0.00152707 | 150 | 0 |
| GAPDH | 189 | C | A | 0.007424282 | 0.001254717 | 150 | 0 |
| GAPDH | 198 | G | A | 0.001933404 | 0.001119903 | 150 | 0 |
| GAPDH | 202 | G | T | 0.009591249 | 0.0009442 | 150 | 0 |
| GAPDH | 226 | G | T | 0.001563921 | 0.00066533 | 150 | 0 |
| GAPDH | 233 | T | G | 0.003640317 | 0.0008913 | 150 | 0 |
| GAPDH | 237 | G | T | 0.004137745 | 0.000637713 | 150 | 0 |
| GAPDH | 238 | G | T | 0.006191293 | 0.00071117 | 150 | 0 |
| GAPDH | 241 | G | T | 0.002015677 | 0.00062742 | 150 | 0 |
| GAPDH | 248 | G | T | 0.000933341 | 0.00060585 | 150 | 0 |
| GAPDH | 265 | G | T | 0.001503991 | 0.000715753 | 150 | 0 |
| GAPDH | 267 | G | T | 3.40E-05 | 0.000696117 | 150 | 0 |
| GAPDH | 270 | G | T | 3.74E-05 | 0.000768497 | 150 | 0 |
| GAPDH | 271 | G | T | 0.001183342 | 0.000613543 | 150 | 0 |
| GAPDH | 288 | G | T | 0.002284136 | 0.000542937 | 150 | 0 |
| GAPDH | 289 | G | T | 0.004813459 | 0.001020567 | 150 | 0 |
| GAPDH | 295 | G | T | 0.000373904 | 0.000856603 | 150 | 0 |
| GAPDH | 302 | C | A | 0.000311101 | 0.00080305 | 150 | 0 |
| GAPDH | 303 | C | A | 0.005530006 | 0.000774617 | 150 | 0 |
| GAPDH | 309 | C | A | 0.001722759 | 0.000829987 | 150 | 0 |
| GAPDH | 329 | C | A | 0.009345466 | 0.00091315 | 150 | 0 |
| GAPDH | 344 | A | C | 0.003568463 | 0.00051852 | 150 | 0 |
| GAPDH | 349 | A | C | 0.000872587 | 0.001024143 | 150 | 0 |
| GAPDH | 357 | C | A | 0.000302393 | 0.0005487 | 150 | 0 |
| GAPDH | 366 | C | A | 6.08E-05 | 0.000707983 | 150 | 0 |
| GAPDH | 383 | C | A | 9.80E-06 | 0.000966213 | 150 | 0 |
| GAPDH | 398 | G | T | 0.008274377 | 0.00052167 | 150 | 0 |
| GAPDH | 399 | G | T | 0.009422908 | 0.000595493 | 150 | 0 |
| GAPDH | 401 | C | A | 1.57E-06 | 0.001083677 | 150 | 0 |
| GAPDH | 407 | G | T | 0.007803253 | 0.00068569 | 150 | 0 |
| GAPDH | 431 | C | T | 0.002383047 | 0.000678163 | 150 | 0 |
| GAPDH | 433 | G | T | 3.34E-06 | 0.000932647 | 150 | 0 |
| GAPDH | 436 | G | T | 0.006427794 | 0.000677103 | 150 | 0 |
| GAPDH | 444 | G | T | 0.00081903 | 0.000823507 | 150 | 0 |
| GAPDH | 454 | G | T | 0.004096618 | 0.00097129 | 150 | 0 |
| GAPDH | 471 | G | T | 0.001290322 | 0.000716023 | 150 | 0 |
| GAPDH | 475 | G | T | 0.003757976 | 0.000674753 | 150 | 0 |
| GAPDH | 522 | C | A | 0.001419561 | 0.000660113 | 150 | 0 |
| GAPDH | 526 | G | T | 0.00367728 | 0.000758763 | 150 | 0 |
| GAPDH | 530 | C | A | 0.008015472 | 0.000689417 | 150 | 0 |
| GAPDH | 536 | C | A | 0.002446945 | 0.000700477 | 150 | 0 |
| GAPDH | 574 | C | A | 0.002741132 | 0.00078885 | 150 | 0 |
| GAPDH | 579 | G | T | 0.000181819 | 0.00057472 | 150 | 0 |
| GAPDH | 606 | C | A | 0.000509095 | 0.000892193 | 150 | 0 |
| GAPDH | 607 | C | A | 0.00165892 | 0.0009289 | 150 | 0 |
| GAPDH | 627 | C | A | 0.000469924 | 0.000613793 | 150 | 0 |
| GAPDH | 652 | C | A | 0.003821338 | 0.000697903 | 150 | 0 |
| GAPDH | 666 | G | T | 0.001298896 | 0.00081307 | 150 | 0 |
| GAPDH | 689 | G | T | 0.004813328 | 0.000919893 | 150 | 0 |
| GAPDH | 713 | T | G | 0.002275224 | 0.001066203 | 150 | 0 |
| GAPDH | 723 | G | T | 0.000153021 | 0.000581653 | 150 | 0 |
| GAPDH | 732 | G | T | 0.001193696 | 0.000828313 | 150 | 0 |
| GAPDH | 738 | G | T | 1.42E-05 | 0.000621793 | 150 | 0 |
| GAPDH | 747 | G | T | 0.002869611 | 0.000836583 | 150 | 0 |
| GAPDH | 761 | C | A | 0.004345526 | 0.000590433 | 150 | 0 |
| GAPDH | 766 | C | A | 0.001768566 | 0.000739507 | 150 | 0 |
| GAPDH | 798 | C | A | 0.004946337 | 0.001136867 | 150 | 0 |
| GAPDH | 799 | C | A | 0.000193331 | 0.00057936 | 150 | 0 |
| GAPDH | 807 | A | C | 0.009049566 | 0.00093734 | 150 | 0 |
| GAPDH | 809 | C | A | 0.001886894 | 0.00064221 | 150 | 0 |
| GAPDH | 825 | C | A | 0.001421896 | 0.000872987 | 150 | 0 |
| GAPDH | 849 | A | C | 0.003005886 | 0.000866567 | 150 | 0 |
| GAPDH | 851 | C | A | 0.005646221 | 0.000626347 | 150 | 0 |
| GAPDH | 861 | A | C | 0.002017133 | 0.001144367 | 150 | 0 |
| GAPDH | 870 | C | A | 0.007923646 | 0.000734437 | 150 | 0 |
| GAPDH | 874 | C | A | 0.002911371 | 0.00072567 | 150 | 0 |
| GAPDH | 885 | C | A | 5.86E-05 | 0.000961373 | 150 | 0 |
| GAPDH | 890 | A | G | 0.001818898 | 0.000502787 | 150 | 0 |
| GAPDH | 892 | C | A | 0.0016496 | 0.000935683 | 150 | 0 |
| GAPDH | 902 | C | A | 0.007406416 | 0.00057353 | 150 | 0 |
| GAPDH | 908 | A | C | 0.008721461 | 0.0010974 | 150 | 0 |
| GAPDH | 909 | C | A | 0.000194887 | 0.000666597 | 150 | 0 |
| GAPDH | 923 | C | A | 0.000707677 | 0.000510553 | 150 | 0 |
| GAPDH | 926 | A | C | 0.005988779 | 0.001868857 | 150 | 0 |
| GAPDH | 934 | A | G | 0.001285891 | 0.001002173 | 150 | 0 |
| GAPDH | 961 | C | A | 0.004335564 | 0.00068364 | 150 | 0 |
| GAPDH | 969 | C | A | 0.00601434 | 0.000649273 | 150 | 0 |
| GAPDH | 971 | A | C | 0.008840072 | 0.001058377 | 150 | 0 |
| GAPDH | 984 | C | A | 0.001768255 | 0.000618943 | 150 | 0 |
| GAPDH | 1011 | C | A | 0.000346337 | 0.000838917 | 150 | 0 |
| GAPDH | 1013 | A | C | 0.008403987 | 0.000842437 | 150 | 0 |
| GAPDH | 1014 | C | A | 0.000157935 | 0.000759147 | 150 | 0 |
| GAPDH | 1019 | A | C | 0.006316856 | 0.000986647 | 150 | 0 |
| GAPDH | 1032 | C | A | 0.002376305 | 0.00067031 | 150 | 0 |
| GAPDH | 1035 | C | A | 0.009235205 | 0.00092209 | 150 | 0 |
| GAPDH | 1040 | C | A | 0.000884302 | 0.000935707 | 150 | 0 |
| GAPDH | 1041 | C | A | 0.00129712 | 0.000785733 | 150 | 0 |
| GAPDH | 1049 | C | A | 0.006586809 | 0.000598297 | 150 | 0 |
| GAPDH | 1050 | C | A | 0.00306528 | 0.000888543 | 150 | 0 |
| GAPDH | 1052 | C | A | 0.003178927 | 0.00092694 | 150 | 0 |
| GAPDH | 1053 | C | A | 0.007165256 | 0.000935107 | 150 | 0 |
| GAPDH | 186 | C | A | 0.000771974 | 0.00062955 | 100 | 20 |
| GAPDH | 195 | C | A | 0.00048631 | 0.00102249 | 100 | 0 |
| GAPDH | 197 | A | T | 0.005442859 | 0.00090923 | 100 | 0 |
| GAPDH | 198 | G | A | 0.009029669 | 0.00090828 | 100 | 0 |
| GAPDH | 199 | T | A | 0.00253865 | 0.000515477 | 100 | 0 |
| GAPDH | 926 | A | C | 0.001948602 | 0.000811323 | 100 | 0 |
